# Functional models from limited data: A parametric and multimodal approach to anatomy and 3D kinematics of feeding in basking sharks (*Cetorhinus maximus*)

**DOI:** 10.1002/ar.25693

**Published:** 2025-06-09

**Authors:** Tairan Li, Mike Schindler, Martha Paskin, Venkata A. Surapaneni, Elliott Scott, Sabine Hauert, Nicholas Payne, David E. Cade, Jeremy A. Goldbogen, Frederik H. Mollen, Daniel Baum, Sean Hanna, Mason N. Dean

**Affiliations:** ^1^ The Bartlett School of Architecture University College London London UK; ^2^ Department of Infectious Diseases & Public Health City University of Hong Kong Kowloon Tong Hong Kong SAR China; ^3^ Department of Visual and Data‐Centric Computing Zuse Institute Berlin Germany; ^4^ Department of Clinical Neuroscience Karolinska Institutet Stockholm Stockholm Sweden; ^5^ Morphik Altmatter Pvt Ltd Krishna Andhra Pradesh India; ^6^ School of Engineering Mathematics and Technology University of Bristol Bristol UK; ^7^ School of Natural Sciences Trinity College Dublin Dublin Ireland; ^8^ Hopkins Marine Station Stanford University Stanford California USA; ^9^ Elasmobranch Research Belgium Bonheiden Belgium; ^10^ Department of Biomaterials Max Planck Institute of Colloids & Interfaces Potsdam Germany; ^11^ Centre for Nature‐Inspired Engineering City University of Hong Kong Kowloon Tong Hong Kong SAR China

**Keywords:** 3D modeling, *Cetorhinus maximus*, digital twin, elasmobranch, filter feeding, linkage biomechanics, wild animal photogrammetry

## Abstract

Basking sharks, *Cetorhinus maximus* (Gunnerus, Brugden [Squalus maximus], Det Kongelige Norske Videnskabers Selskabs Skrifter, 1765, vol. 3, pp. 33–49), feed by gaping their mouths and gill slits, greatly reorienting their cranial skeletons to filter food from water. The 3D biomechanics of this behavior, however, are exceptionally difficult to study due to the size, elusiveness, and CITES status of these animals and the rarity of well‐preserved specimens. To overcome these challenges, we integrated anatomical, digital design, and computer imaging approaches to reconstruct bio‐realistic and poseable 3D skeletal models of feeding basking sharks. The skeleton, segmented from CT scans of intact heads, was first abstracted as a rigging for guiding skeletal positioning in 3D space. Directed by the anatomies of museum specimens and dissected beached animals, the digital scaffolding was used to virtually correct skeletal distortions (e.g., from specimen collapse), resetting the skeleton to closed‐mouth symmetry. Open‐mouthed feeding postures were recreated by repositioning skeletal joints to biologically relevant destination coordinates defined from videos of feeding sharks, exploiting the basking shark's steady feeding posture to build 3D photogrammetry models from successive video frames. The resultant “digital puppet” bridges diverse imaging data while capturing the coordinated motion of “hidden” cranial joints, deconstructing complex form‐function relationships into computationally controllable parameters for exploring 3D skeletal movement. The input data gathered for our model provides new perspectives on basking shark cranial anatomy, while the model's biological fidelity gives insights into dynamic feeding processes impossible to observe in the laboratory. Branchial arch mechanics are comparatively poorly studied in sharks; our model can act as a platform for future kinematic modeling (e.g., of individual variation, other species), while demonstrating interdisciplinary approaches for studying large and elusive wildlife.

## 1 INTRODUCTION

The largest vertebrates occupy vast oceanic habitats, traverse long distances, and dive to extraordinary depths, which collectively make observing movements and behaviors extremely challenging. Studies must contend with the logistical obstacles associated with long observation periods, rough seas, inclement weather, and limited visibility underwater. In addition, study species are often endangered, elusive, and occurring in small populations, impeding discovery and monitoring. The protection status of endangered species (e.g., CITES, CMS, OSPAR) demands particular precautionary measures (e.g., keeping distance, responsibility to not harm), further hampering close observation (Rigby et al., 2021). Since the appearance at the water surface is unpredictable and the tracking of animals in 3‐dimensional vertical habitats is complicated, diverse technology is needed to determine species' whereabouts (e.g., ships, air‐ or water‐borne drones, aircraft, satellites). Because observation data is fundamental to characterize animal ecology, behavior, and physiology (e.g., drivers of activity, physiological constraints, migration patterns), biologging tags are currently state‐of‐the‐art equipment for studies of marine megafauna (Goldbogen, Cade, Boersma, et al., 2017; Watanabe & Papastamatiou, 2023).

Although modern biologging tags and animal‐borne recording devices can be customized for various types of data collection (e.g., with cameras, GPS, gyroscopes), most cannot provide the tissue and in vivo physiology data necessary for biomechanical analyses of living animals (Fahlman et al., 2021). Unfortunately, carcasses washed up on land end up being a primary source for studying the anatomy of large marine animals, beyond the use of basic image data or biopsies. Accordingly, the materials for anatomical descriptions of marine megafauna are typically incomplete and/or heavily distorted: stranded specimens are often damaged due to boat‐strike and beaching, from their heavy bodies collapsing on land, and/or from scavengers and microbial decomposition occurring rapidly in marine megafauna, due to large body size and fat proportions (Christiansen et al., 2019; da Cunha Ramos et al., 2024; Janaway et al., 2009; Moore et al., 2020). Marine megafauna therefore present unique logistical challenges for in vivo observations, tissue harvesting, sample transport, and high‐resolution 3D data acquisition, obstacles that are irrelevant for smaller taxa (Christiansen et al., 2019; Segre et al., 2023; Shero et al., 2021).

The obstacles facing anatomical study of marine megafauna are especially applicable to suspension filter‐feeding vertebrates, many of which, across taxonomic groups, evolved massive sizes (Stiefel, 2021). Studies on filter‐feeding elasmobranchs (sharks and rays) are particularly rare in comparison to the anatomical, biomechanical, and materials work on filter‐feeding whales (Mysticeti) (e.g., Christiansen et al., 2019; Fudge et al., 2009; Segre et al., 2023; Werth & Potvin, 2016). Filter‐feeding sharks and rays, however, are particularly interesting for exploring the morphological evolution of filter feeding as a foraging specialization. Not only did filter feeding apparently arise multiple times independently among elasmobranchs, the structures and behaviors involved differ considerably (Friedman, 2012; Friedman et al., 2010; Misty Paig‐Tran & Summers, 2014; Motta et al., 2010; Tomita et al., 2011). Additionally, elasmobranch filter feeders have smaller maximum sizes than filter‐feeding whales, making them considerably more tractable for close observation (Motta et al., 2010). Yet, beyond generalized kinematic observations and inferences of feeding behavior (e.g., Montero‐Quintana et al., 2021; Motta et al., 2010; Nakaya et al., 2008; Tomita et al., 2011), data on feeding biomechanics of elasmobranch filter feeders remain limited and technically challenging to obtain directly, although digital and physical simulations of feeding structures have given important insights into filter hydrodynamics (e.g., Divi et al., 2018; Paig‐Tran et al., 2011; Sanderson et al., 2016).

**FIGURE 1:**
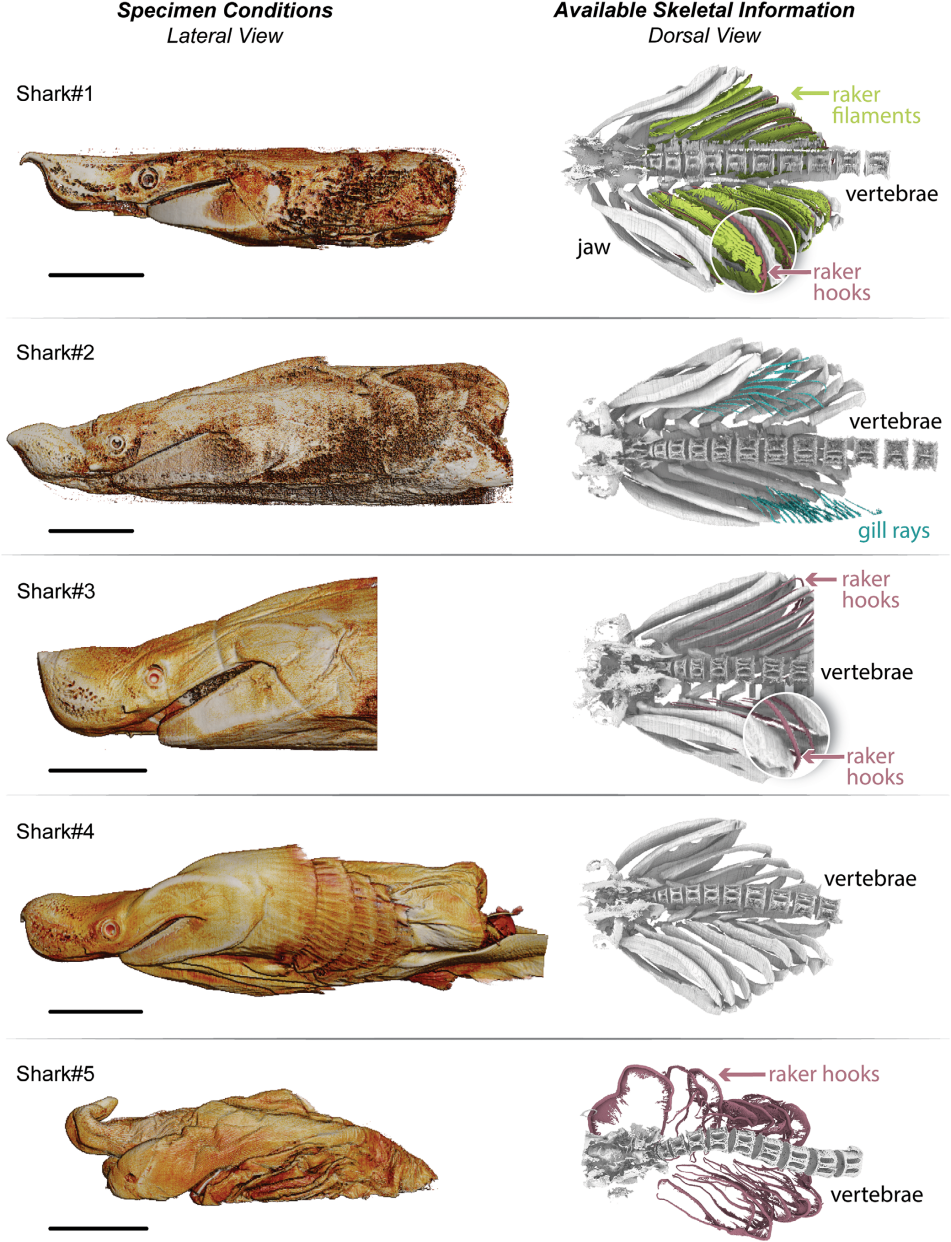
Volume renderings of medical CT scan data for Sharks#1–5. The left column shows the external appearance in left lateral view, while the right column shows the inner skeletal structure in dorsal view. All specimens were scanned with their mouths closed: Note that the consecutive series of visceral arches have a ‘brushed back’ configuration, all being roughly parallel and angled caudo‐laterally. There was great variation in specimen size, scan/specimen completeness, and condition; as a result, some features are more visible in some scans than others (e.g., structures indicated by colored text in the right column). Scale bars = 20 cm. See Figure 4 for more detail regarding gill raker hooks and filaments.

**FIGURE 2:**
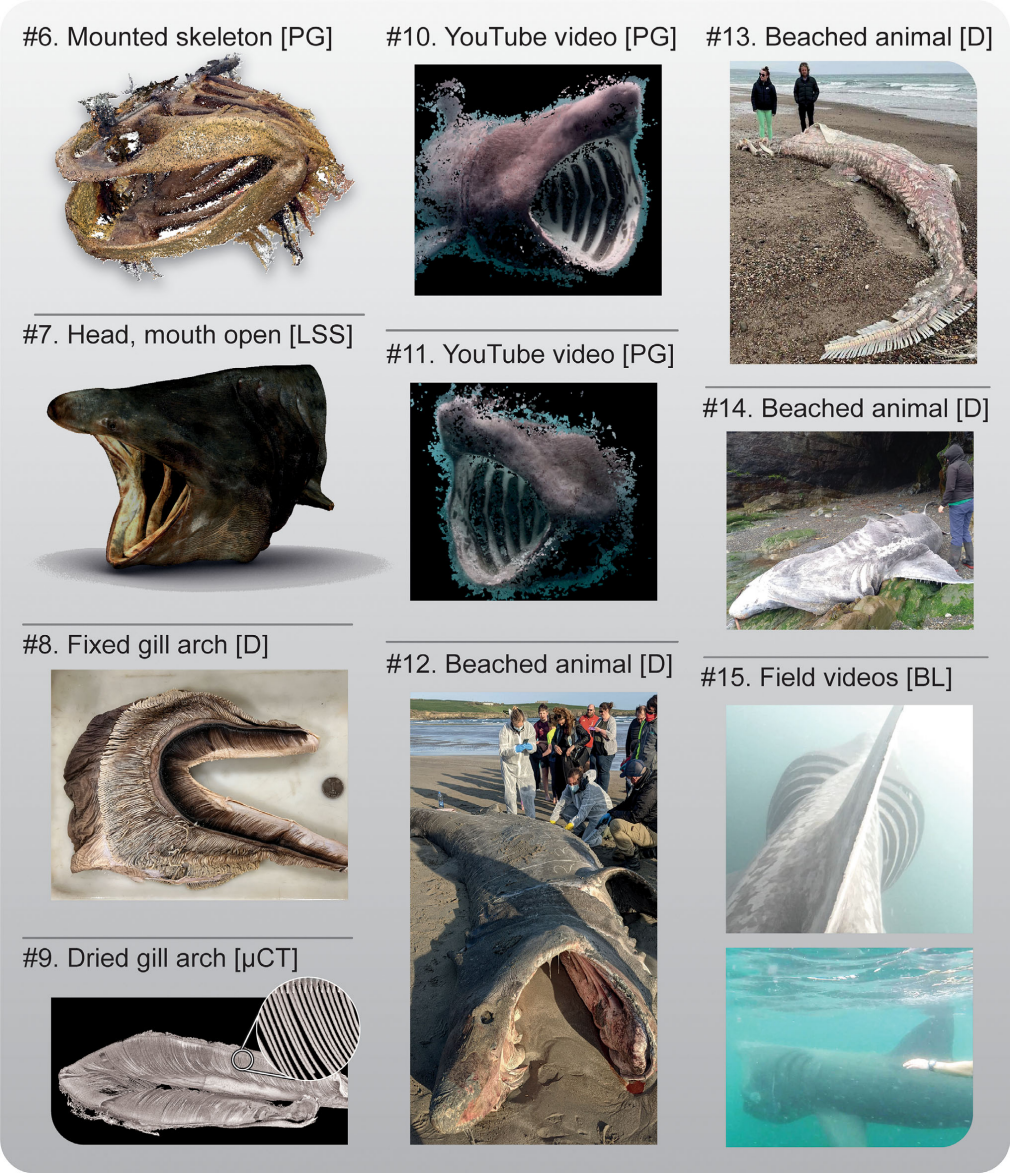
Additional data sources used for informing anatomical reconstructions. Since individual specimens varied in quality, preparation, and completeness, information from all specimens was combined for a more unified picture of basking shark anatomy. See Table S1 for more information on individual specimens. Bracketed letters following specimen descriptions indicate the nature of the specimen: Biologging tag videos (BL), dissection (D), laser surface scan (LSS), microCT (μCT), photogrammetry (PG).

**FIGURE 3:**
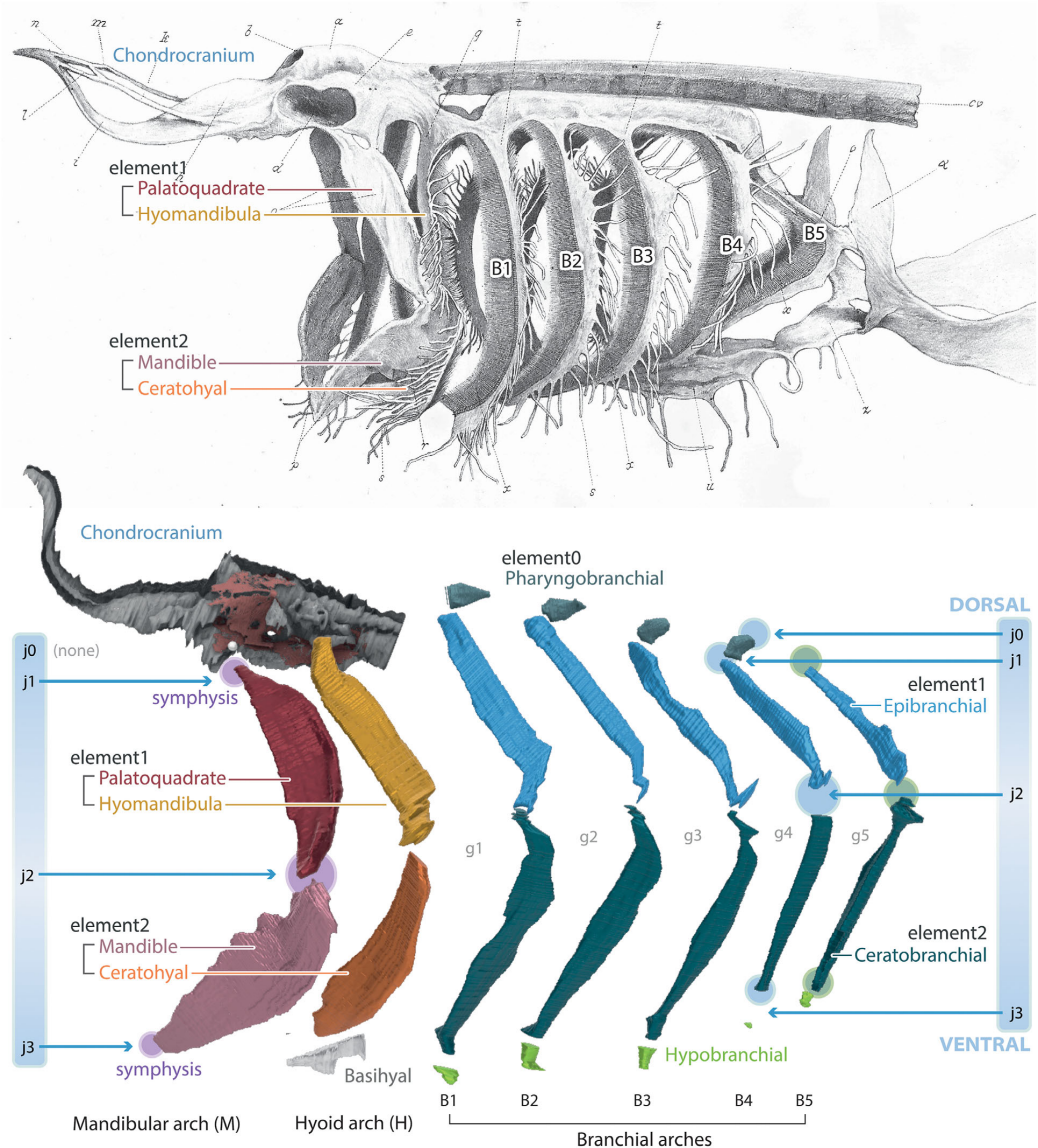
Comparison of the existing most complete drawing of basking shark skeletal cranial anatomy (Pavesi, 1874; top image) with segmented medical CT data from Shark#1 (bottom image). Labels in the bottom image illustrate the element and joint nomenclature used in the current study, with only elements on the animal's left side illustrated for clarity; right side elements, the vertebral column, and many ventral elements (e.g., basibranchials) are excluded for clarity. Several elements in Pavesi's image are similarly labeled to facilitate comparison. The dorsal and ventral elongate elements in each arch (*element1* and *element2*, respectively) are linked by a lateral joint (*j2*) with the more dorsal (*j1*) and ventral joints (*j3*) connecting the elongate elements to the remainder of the branchial basket. An additional, more proximal dorsal midline joint (*j0*) is present only in some arches (see text). The distortions at the ends of the elongate elements are a function of CT scan resolution and poor mineralization of end regions, challenging segmentation. The locations of internal gill slits (*g1*–*g5*) are indicated; compare with Pavesi's illustration, which also shows the general position of gill raker sheets relative to the gill slits (see also Figure 4). Note how individual arch elements (e.g., *element1* and *element2* in the hyoid arch) cannot be distinguished in Pavesi's image, indicating the specimen was defleshed, but left with connective tissue still sheathing the skeleton. Arch abbreviations in the bottom image (e.g., Mandibular arch: M; Hyoid arch: H; Branchial arches: B) are also used in later figures. Dashed lines and cursive letters in the top image are maintained from Pavesi's original illustration and are not detailed here.

**FIGURE 4:**
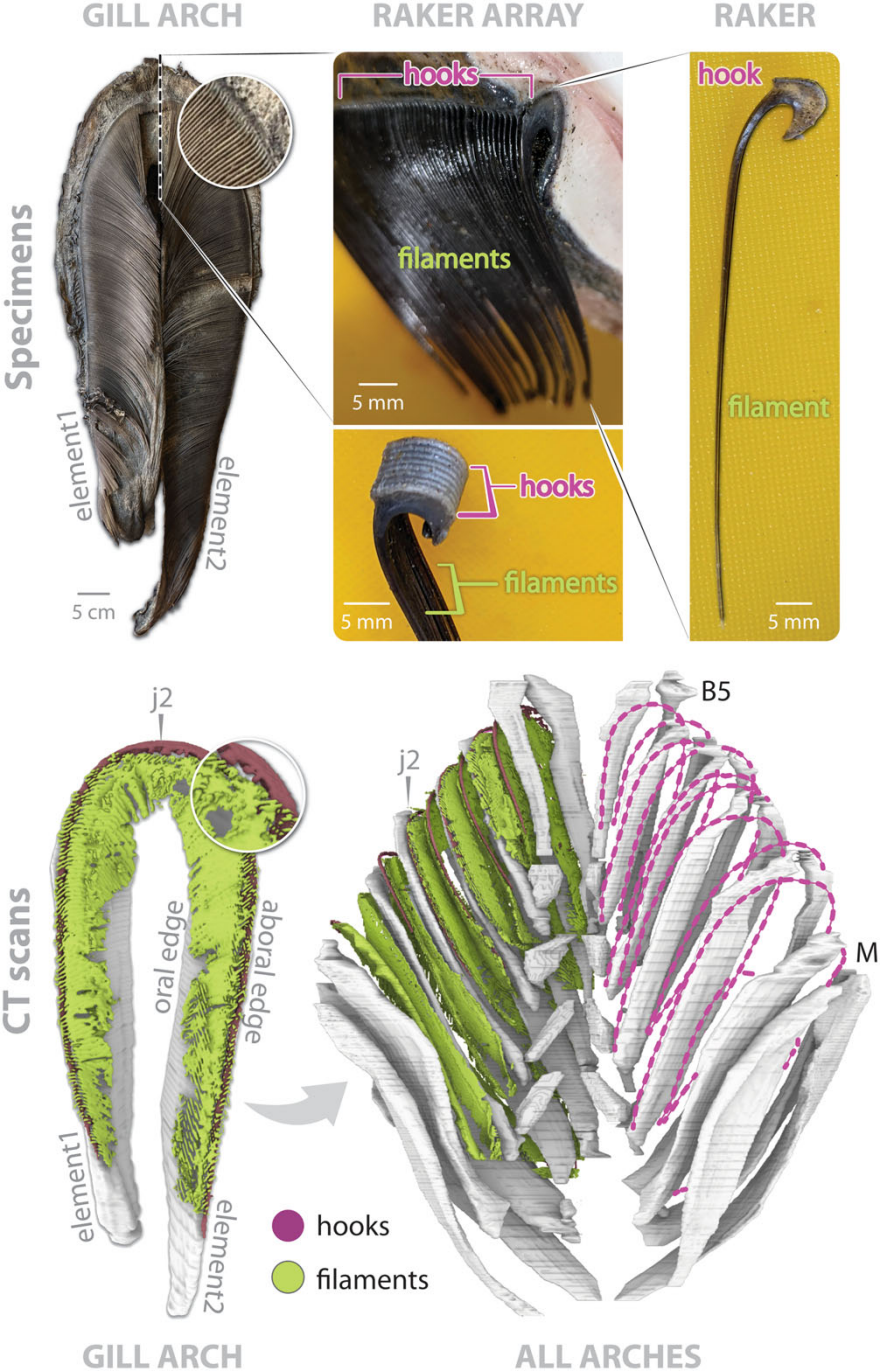
Gill rakers and their arrangements in arrays relative to the visceral arch skeleton, shown in dried/wet specimens in the top row and CT scans on the bottom row of images. Gill rakers can cover both sides (branchial arches 1–4) or just one side (hyoid arch, branchial arch 5) of intact visceral arches (e.g., top row, “Gill arch” image; see Figure 3 for arch anatomy). Individual gill rakers (e.g., “Raker” image) possess blade‐like hooks at their bases, where they are anchored into tissue, tapering to elongate filaments, extending into the water. Rakers are arranged in long series, with their hooks stacked and bound together (“Raker array” images), tracking the aboral margins of visceral arches (i.e., the margin not bordering the pharynx), with their filaments extending toward the oral side of each visceral arch and into the pharynx. In the dried museum specimen (upper left; Shark#9), with its medial edge oriented downwards in the image, the gill rakers are visible as a continuous fringe, with the tips of the raker filaments pointing toward the oral (interior) margin of the arch. Note that raker array is not interrupted at the joint between the gill arch's two elongate elements (*j2* between *elements 1* + *2*, see Figure 3). Since the stacked arrays of hooks are more stout than the raker filaments, in CT scans (bottom row) the hook arrays were often clearly visible as continuous arcs between arches, even when raker threads were not resolvable. For example, in the CT scans of an individual arch (bottom row: “Gill arch” image) and an entire head skeleton (“All arches”) shown here, the entire intact hook array (purple color) could be segmented from the patchy sheet of gill filaments (green color). In such cases, the hook array was traceable using Amira's *Autoskeleton* module (dashed purple lines in the bottom right image). The gill arch images in the top and bottom rows are in comparable anatomical orientations, while the cranial skeleton on the bottom right is shown in dorsal view.

Basking sharks (*Cetorhinus maximus*; Figures 1 and 2) are obligate feeders on zooplankton and the largest mackerel sharks (Dolton et al., 2023), showing seasonal migrations that can result in aggregations of hundreds of feeding individuals at certain times of year (Gore et al., 2023; Sims, 2008). Unlike rorqual whales, which “lunge feed” by intermittently accelerating through, engulfing and filtering large volumes of prey‐laden water (Goldbogen et al., 2017; Simon et al., 2012), basking sharks “ram feed” by swimming slowly (~ 0.85 m/s), holding their huge mouths open (e.g., Figure 2, Shark#15) for minutes at a time to accumulate zooplankton, mainly copepods (Matthews & Parker, 1950; Sims, 2008). Continuous ram filter feeding using slow speeds (<1 m/s) is more similar to the foraging strategy of bowhead and right whales (Simon et al., 2009; van der Hoop et al., 2019; Werth, 2004), whale sharks (Cade et al., 2020), and some suspension‐feeding fish (Carey & Goldbogen, 2017; Sanderson et al., 1994). However, unlike the fringed and matted baleen filter of mysticete whales, hanging from the top of the mouth (Fudge et al., 2009; Szewciw et al., 2010; Werth, 2013), the basking shark filter comprises thousands of elongated structures (gill rakers) arranged in comb‐like rows (Figures 3 and 4) at the lateral exits of the pharynx (Figures 1 and 3) (Gross‐Lerner, 1957; Schnakenbeck, 1955; Turner, 1880).

The gill rakers of basking sharks are unmistakable structures, recognizable in the fossil record for over 40 million years (Cione & Reguero, 1998; Prokofiev & Sychevskaya, 2018; Welton, 2013a, 2013b). Individual gill rakers comprise a blade‐like hook proximally, where they are anchored into the tissue of the pharynx, tapering into a thread‐like filament (Figure 4), likely extended into the flow when basking sharks are feeding (although this has never been shown explicitly). Although the precise 3D positioning and tissue associations of gill rakers have not been clearly demonstrated in the literature, it is clear that the gill rakers are arrayed along the branchial arch cartilages (Figures 3 and 4), rod‐ and plate‐like skeletal elements connected by joints in series to form the “branchial basket” that supports the throat (oropharynx) (Figures 3 and 4) (Matthews & Parker, 1950). The connected joints of the branchial basket seem to allow the throat skeleton of sharks to expand and collapse to some degree, providing control of water movement during feeding and ventilation (van Meer et al., 2019; Wegner, 2015). In basking sharks, however, branchial basket expansion during feeding appears to be more extreme, with the mouth and branchial region ballooning outward as the animal opens its massive gape (Figure 2, Sharks#10–11). Yet, the large size and swimming speed of these animals has made their feeding biomechanics challenging to study, particularly with regard to how the skeleton and the gill rakers interact and move during feeding.

Here, we use a novel integration of anatomy and design approaches to examine feeding biomechanics in the basking shark, combining CT scanning, photogrammetry, biologging, and underwater video data, and parametric modeling. The combined method generates a poseable parametric model of the basking shark head skeleton, using behavioral data of live animals to guide registration of skeletal data. Results provide novel perspectives on basking shark functional anatomy, while framing new methods for studying skeletal motion in marine megafauna. Our overall approach is necessarily multi‐disciplinary with diverse techniques connected in feedback loops, where results from each tool or observation provide inputs for others. We therefore present the work in a non‐linear and non‐traditional structure; for example, some of the findings that guide our methodology are outlined in the Materials & Methods rather than as standard “results”. Additionally, since the parametric model devised is one of the primary outputs of the work, we do not use the combined Results & Discussion section to recap this result, but rather to frame how it can be used, detailing its broader implications with regard to techniques, anatomy, and feeding biomechanics.

## 2 MATERIALS AND METHODS

**FIGURE 5:**
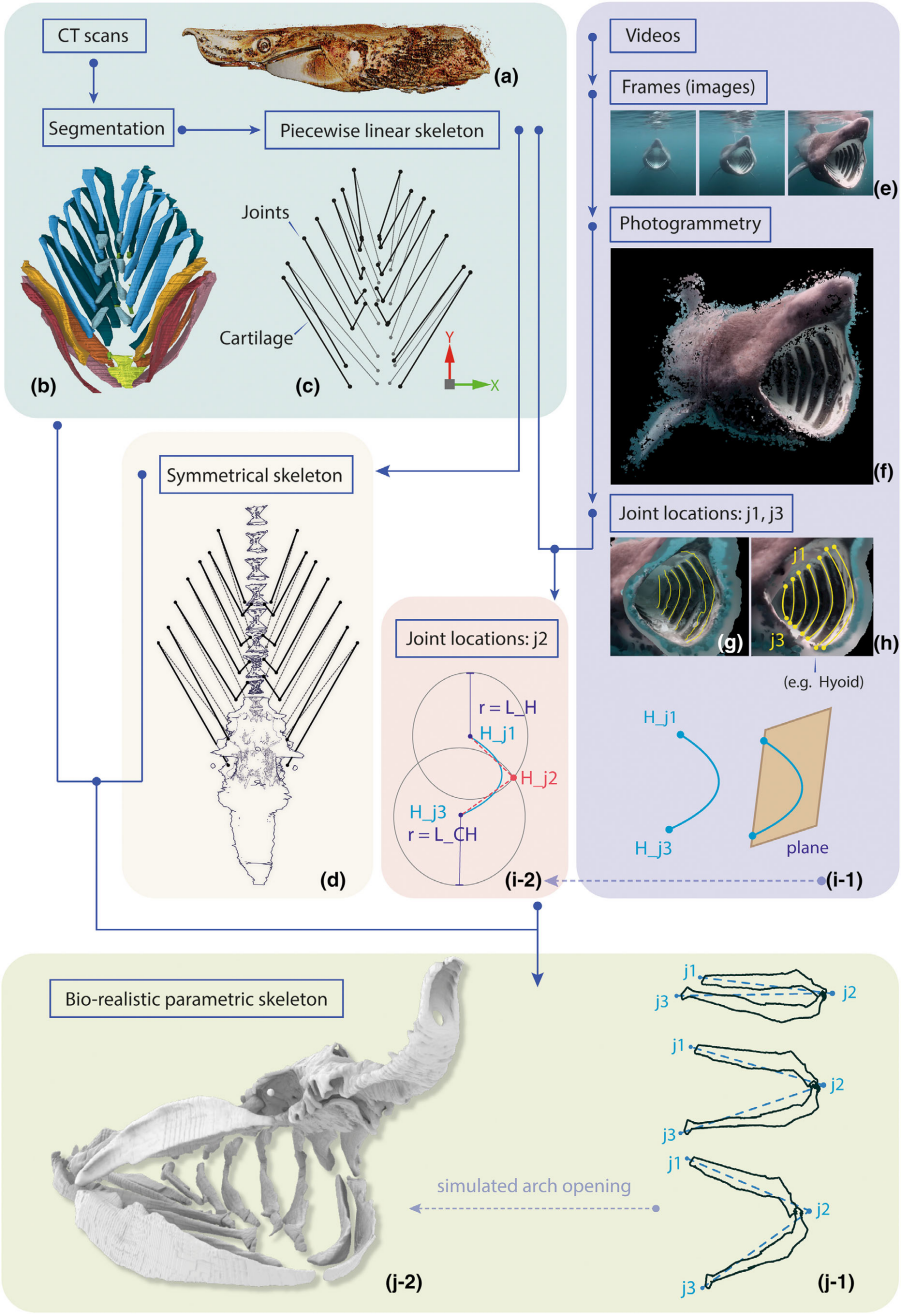
Flow chart of methodology used in the current study. (a) CT scanning provides digital anatomical information that can be (b) segmented to individual skeletal elements, which then can be used to (c) construct a poseable piecewise linear skeleton, for (d) restoring biological symmetry and downstream mimicry of kinematic poses (see below). (e) Using stills from videos showing feeding sharks from multiple perspectives, (f) 3D models of feeding sharks were constructed. From these, (g) arches were iteratively traced (see text), then (h) cleaned as smoothed (i‐1) plane curves, providing the locations for the dorsal and ventral joints framing the elongate elements (*j1* and *j3*, respectively). (i‐1) Using those joint positions and (i‐2) element lengths derived from CT data, the range of plausible locations for the lateral joint (*j2*) are defined, with the position always distanced from *j1* and *j3* according to the corresponding element lengths. (j‐1, j‐2) The resultant joint positions provide the destination coordinates for actuating the linear skeleton to the open‐mouthed feeding position, where the original meshed skeletal elements can be placed to render the bio‐realistic skeleton. L_H, length for hyoid; L_CH, length for ceratohyal; H_j1, Hyoid arch joint#1.

Our approach for characterization and modeling of the basking shark feeding mechanism (Figure 5) involves reconstruction of anatomy from diverse sources (CT scans, surface scan of an intact head, photogrammetry of a skeletonized head, beached animal dissections), guided by control parameters derived from observations of live animals (models reconstructed from video‐based photogrammetry, field videos of feeding behavior) (Figures 1 and 2). The varied data sources, relevant data and specimen parameters, and analysis tools used are detailed in Table S1.

### 2.1 Specimen CT scans

To visualize internal skeletal anatomy, medical CT scans of five basking shark heads were performed by the authors or collected from other sources (Figure 1 and Table S1). Scans were reconstructed at the scanning location and exported as image stacks (e.g., DICOMs), then analyzed and visualized in Amira software (Amira ZIB Edition, v. 2021.30).

Given our interest in the feeding mechanism, we focused exclusively on the cranial skeleton, including the seven visceral arches (the jaw/mandibular, hyoid and five branchial arches), as well as the chondrocranium (braincase) and anterior portion of the vertebral column (Figure 3). The anatomical information available from scans varied considerably (Figure 1), due to variable fixation quality and specimen condition, specimen deformation (e.g., distorted under their own weight), and incompleteness of the specimen or scan (e.g., the scan from Shark#3 stopped anterior to the end of the branchial basket). As a result, specimens proved useful for different information. For example, Sharks#1–4 showed reasonable preservation of the chondrocranium, with additional elements variably distinguishable, including branchial rays (Shark#2, colored blue); the entire population of gill rakers (Shark#1, colored green) or merely their hooks (Sharks#3,5, colored red); and vertebrae (Sharks#1–5). In some scans, elements were missing or indistinguishable; for example, the branchial basket was incomplete in Shark#3, and almost no skeletal information was discernible in the heavily deformed Shark#5. Despite Shark#5 being crumpled and poorly preserved, its populations of gill rakers were visible, as well as their relationships to other structures (e.g., gill slits, pharynx).

None of the scans clearly showed all cranial elements together. As information on the varied appearance of relevant cranial structures in CT had not been previously published, the combined data from the multiple scans was therefore particularly useful in identifying features and their anatomical relationships for downstream kinematic models. Where possible, individual skeletal elements were segmented (digitally dissected) by a combination of thresholding and manual segmentation in Amira ZIBEdition 2021.30 (Paskin et al., 2022). Elements of the skeleton were identified according to previous anatomical descriptions (Pavesi, 1874, 1878; Senna, 1925); the top image in Figure 3  shows Pavesi's original 1874 illustration. The ‘resting’ positions (i.e., non‐feeding positions) and interactions of skeletal elements were verified in dissections of beached individuals (Figure 2 and Table S1: Shark#12–14). Clarification of anatomical relationships was also supported by physical (Shark#6‐9) and photogrammetry investigation (Shark#6) of anatomical specimens, including a unique skeletonized specimen housed at the Zoological Institute, Kiel University, Germany (Shark#6), mounted on a supporting metal frame and therefore not suitable for CT scanning. The hook portion of the gill raker array, once identified (see *Results/Discussion*), was traced using the ‘*Auto Skeleton’* module in Amira ZIBEdition 2021.30 (Figure 4, purple curves).

Understanding the relationships among branchial arch elements (Figure 3) and between branchial arches and gill rakers (the primary filter elements; Figure 4) was of highest priority. Scans with complete cranial skeletons and resolution allowing rakers to be distinguished were preferred for guiding downstream skeletal modeling initiatives. Therefore, we used the Shark#1 scan (Figure 1) as the baseline for the majority of subsequent modeling steps (Figure 5a,b).

### 2.2 Restoring biological symmetry in skeletal data using mathematical principles

All CT‐scanned specimens were distorted in some way, shifted relative to their natural/biologically symmetrical alignment (e.g., from collapse under their own weight or fixation in odd positions; Figure 1). In some specimens, for example, although the vertebral column showed little lateral bending and the dorsal body stayed in a relatively convincing position, ventral unpaired elements (i.e., mandibular symphysis, basihyal, basibranchials) were shifted away from the midline. Importantly for our method, we observed that, due to the linkages among elements in the head skeleton, deformations tended to result in associated shifts of paired cartilages (left vs. right elements) (e.g., Figure 4, bottom right image). For example, in a specimen where the branchial basket was twisted counter‐clockwise about the vertebral column when looking from the anterior view, a branchial arch joint on the animal’s right side would be shifted ventrally and medially by a certain distance which, due to the linkage of elements, would result in a concomitant dorsal and lateral displacement of the comparable joint on the animal’s left side.

The cranial skeleton therefore had to be ‘reset’ to its natural alignment before constructing downstream kinematic models. To accomplish this, we started from a technique previously developed by our group (Paskin et al., 2022), abstracting complex skeletal identities as piecewise linear skeletons, with joints represented by points and cartilages as lines between points (Figure 5c). With this approach, Paskin et al. (2022) previously coded for the best‐preserved half of the body (left or right), mirrored it contralaterally (i.e., to the other side), and thereby created an idealized ‘skeletonized’ model, based on only one side of the body. In the current work, we built on this approach and dataset, creating a protocol for parameterising a skeletonized linear model of the complete but distorted cranial skeleton that then could be used to guide deformation corrections in the original CT data.

As in Paskin et al. (2022), the 3D coordinates of the linear skeleton (describing the configuration of skeletal joints in space) were first determined by constructing a ‘*Spatial Graph*’ from segmented skeletal data in Amira software. The list of joint configuration information, including joint coordinates and segment connectivities (e.g., which elements were linked in which order), were exported as .xml and reformatted as .csv in Microsoft Excel, then imported into the Grasshopper plug‐in for Rhinoceros 7 (McNeel & Associates), where all subsequent skeletal codings and reorientation operations were performed (described below).

#### 2.2.1 Modeling assumptions

**FIGURE 6:**
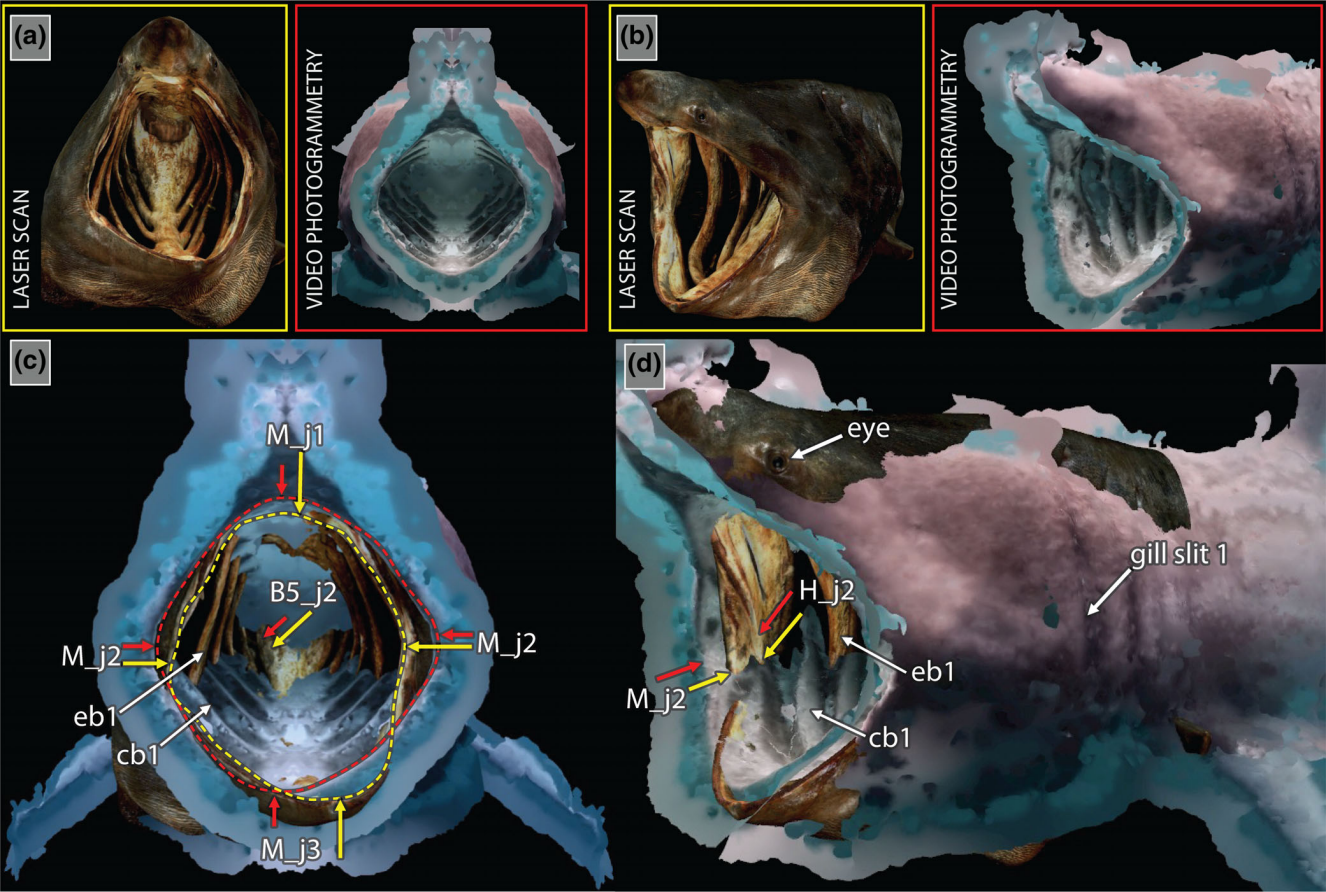
Overlay of the in‐air laser‐scanned model (Shark#7, in yellow) and the photogrammetry in‐water model (Shark#10, in red) at maximum gape, in frontal (a/c) and lateral (b/d) positions. Models were scaled and aligned based on anatomical landmarks (e.g., eyes, gill slits, pectoral fins, tip of rostrum). Dashed lines in (c) approximate mouth outlines and arrows indicate comparable landmarks, colored according to each model's configuration and labeled using the nomenclature in Figure 3. The photogrammetric model captures the mouth geometry of a living shark during feeding and exhibits a larger lateral gape (distance between left and right *M_j2*). The gape of the laser‐scanned museum specimen on the other hand is narrower but deeper, probably due to gravity and deformation (compare positions of *M_j3, mandibular symphysis*). This difference in ventral depression is reflected in all subsequent visceral arches, as evidenced by the floor of the throat of the in‐air model being largely hidden below that of the in‐water model in (c). The more yellow region visible at the caudal end of the pharynx is an indication of the limited movement of the more caudal branchial elements (see also Figure 8). Note the close association of the mandibular (*M*) and hyoid (*H*) arches in (d), which move as a unit. Branchial arch#2 (*B2*), ceratobranchial (*cb2*) epibranchial (*eb2*); branchial arch#5 (*B5*).

**FIGURE 7:**
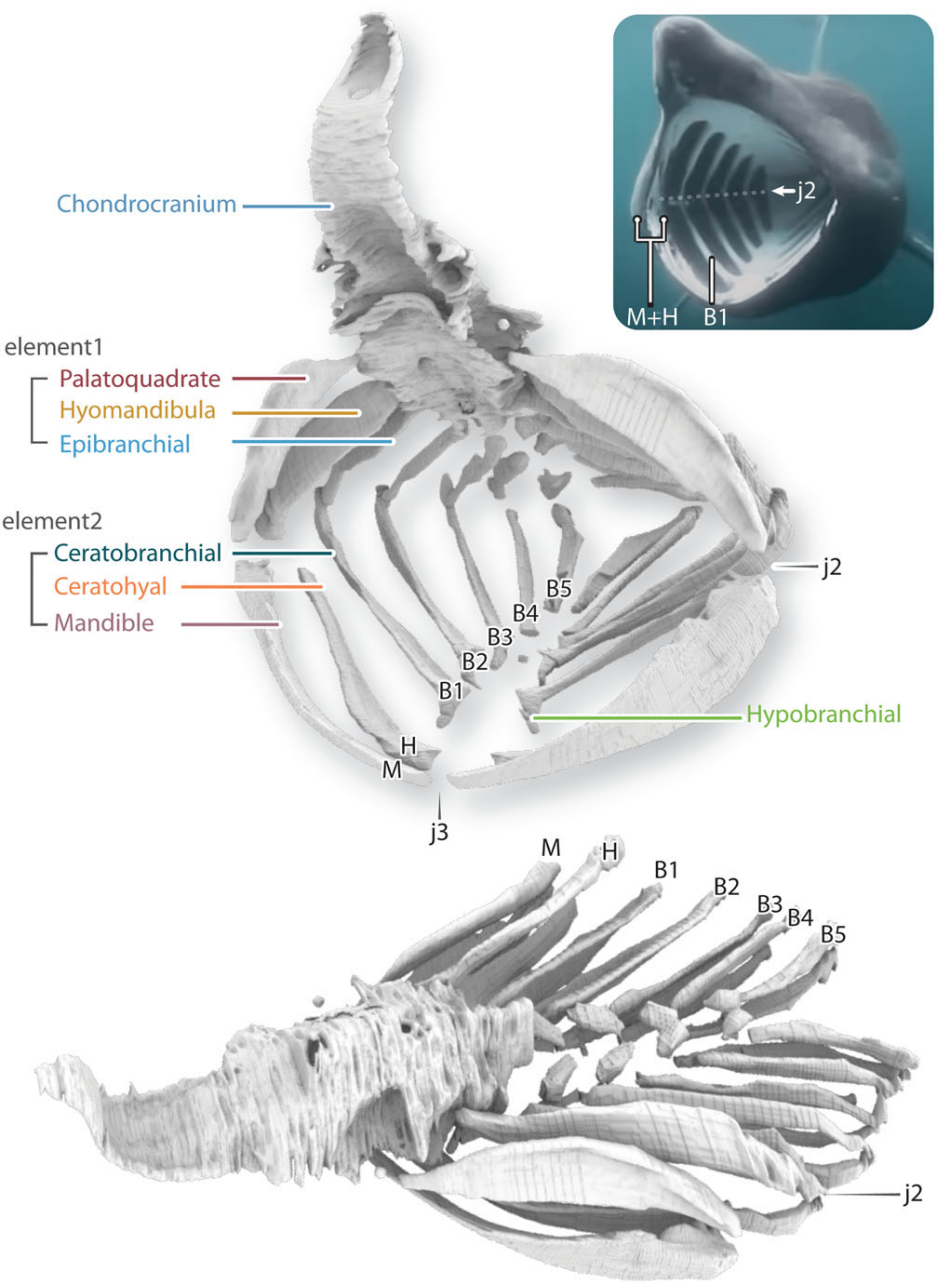
Parametric basking shark skeleton posed in fully‐open configuration (top image, anterolateral view) and closed‐mouth configuration (left dorsolateral view, bottom image). The open‐mouthed model corresponds well with the anatomy of a live feeding shark (inset image), where the branchial arches and their lateral joints (*j2*) are visible in the open gape. The basihyoid and basibranchial elements (forming the ventral floor of the pharynx) are excluded in the model. Note that the mandibular (M) and hyoid (H) arches are bound together by soft tissue and can hardly be distinguished in the live animal. Branchial arches (B) are numbered in rostrocaudal order. This abbreviated visceral arch nomenclature (i.e., M, H, B1‐5) is used for the remaining figures.

For specimen deformation correction, we made several simplifying assumptions based on our anatomical observations of numerous specimens (Table S1):

(a) left–right paired joints (i.e., all joints except the jaw midline symphyses) are, in the living animal, symmetrical about the midline and their positions can be described by coordinates in Cartesian space;

(b) joints and cartilages may be displaced in distorted samples, but skeletal elements do not change their lengths, joint stretch is minimal, and cartilages are effectively inflexible (i.e., the cartilages do not bend and change the Euclidean distance between adjacent joints);

(c) in the living animal, unpaired elements (e.g., chondrocranium, vertebral column, basihyal, basibranchials) and unpaired joints (jaw symphyses) should typically be aligned to the animal's mid‐sagittal plane. In this way, given the animal's bilateral symmetry, we consider that left and right members within each bilateral pair of joints are equidistant from the mid‐sagittal plane and that the chondrocranium and vertebral column sit on the mid‐sagittal plane (Figure 5c);

(d) the longest elements in each visceral arch contribute the most to throat expansion during feeding. This assumption is based on all examined visceral arches containing two particularly elongated cartilages (Figures 3 and 4). These dorsal and ventral elongate elements were verified in dissections, CT scans, and feeding videos to be especially mobile and to frame the pharynx laterally (the dorsalmost element of each pair is listed first in the following arches):

- the palatoquadrate and mandible (jaw arch);
- the hyomandibula and ceratohyal (hyoid arch); and
- the epibranchial and ceratobranchial (in each branchial arch).

(e) concomitantly, smaller elements in series with the elongated elements were considered to be less relevant for understanding feeding movements. The pharyngobranchials (dorsalmost elements in the first to fourth branchial arches; Figures 3 and 4) were observed to be largely embedded in tissue (e.g., Figures 6b and 7 inset) and not particularly mobile. Similarly, the most ventral elements (i.e., basihyal, basibranchials and hypobranchials) were found to be bound together by connective tissue; we noted little relative displacement among elements in this group, although this entire ventral series appeared to move en masse, undergoing a large excursion during feeding (Figures 6b and 7 inset).

We therefore posit that by describing the 3D movements of elongate elements, we can capture the most relevant reconfigurations of cranial elements during feeding.

#### 2.2.2 Coding elements of the skeleton

To facilitate calling grouped elements for applying functions and correcting deformations in visceral arch chains (see below), we coded all visceral arches using the same ordering system (Figure 3). In each arch, the highly mobile elongate elements (see *assumption d*) were numbered [*element1*] and [*element2*] (dorsal and ventral, respectively). Where a smaller, more dorsal element was present (i.e., the pharyngobranchials in the branchial arches B1‐4), this was marked as [*element0*]. The mandibular and hyoid arches and the last branchial arch (B5) contained no individual elements dorsal to [*element1*]. Additionally, the so‐called gill ‘pickaxe’ of B5 (caudal pharyngobranchials fused with the B5 epibranchial; de Carvalho, 1996; Versluys, 1922) was also indiscernible in our scans. Ventral elements (i.e., basihyal, basibranchials) were not numbered in our ordering system, since they are distal in the kinematic chain and our observations of feeding animals and open‐mouthed specimens indicated ventral elements move in concert as a continuous ventral platform in the pharynx (Figures 6b and 7 inset).

Based on our assumptions regarding feeding kinematics (see *assumptions d‐e*), the joints most relevant to feeding movements are those at the ends of the elongate elements; we numbered these [*joints1‐3*], in dorsal to ventral order, with [*joint2*] being the lateralmost joint in the series, between the two elongate elements (*j2* in Figures 3, 4, and 5i, j). With this coding system, [*joints1‐3*] therefore formed a triangular plane describing the arrangement of each arch's elongated elements (Figures 3, 4, and 5i, j). In the branchial arches, an additional [*joint0*] was coded between the pharyngobranchial (i.e., [*element0*], when present) and the axial column (Figure 3).

#### 2.2.3 Establishing the animal's mid‐sagittal plane

To determine the target coordinates for correcting skeletal deformations according to the assumption of bilateral symmetry (see *assumption a* above), we began by defining a mid‐sagittal plane. For each arch, we first averaged the signed coordinates of the left and right [*joint1*] and [*joint3*] instances, respectively (i.e., 14 pairs of left and right joints: 7 pairs of dorsal joints and 7 pairs of ventral joints); this established, for each arch, the average dorsoventral (Z) and rostrocaudal (Y) locations for [joint1] and [joint3] ([*ave1*] and [*ave3*], respectively), near to the body's midline. The mediolateral positions (X‐coordinates) for all arches' [*ave3*] were then averaged again to define a common mid‐sagittal X‐coordinate (X0), establishing the body's mid‐sagittal plane. All points describing each arch (i.e., [*joints0‐3*] on left and right sides) were then translated mediolaterally along the X‐axis to align each arch's [*ave3*] to the newly defined mid‐sagittal coordinate (X0). Each arch was then rotated around its respective mid‐sagittal coordinate in the XZ plane (i.e., the arch's new [*ave3*]) until the X‐coordinates of its [*ave1*] were also aligned with the mid‐sagittal plane (i.e., equalled X0). This registered the 14 midpoints of all arches (the [*ave1*] and [*ave3*] of all seven arches) to the mid‐sagittal plane, defining a common dorsoventral axis for all arches and a mid‐sagittal (YZ) plane to guide the establishment of bilateral symmetry in downstream steps.

#### 2.2.4 Restoring bilateral arch symmetry

In order to restore bilateral symmetry of skeletal positions in deformed specimens (e.g., Figures 4 and 5b,c), we first mirrored the joints of each arch to the opposite side of the body (e.g., right joints mirrored to the left side) using the mid‐sagittal YZ plane defined in the previous step. This resulted in paired sets of point coordinates for each joint on one side of the body (i.e., left). Next, we averaged the unsigned pairs of coordinates for each joint position (i.e., the original position and its mirrored position) to determine the average position for each joint in their new configurations established in Section 2.2.3. The averaging of bilateral joint positions, however, broke the length constraint we previously defined for skeletal elements (see *assumption b* above). To solve this, we first calculated the mean lengths of each left–right pair of arch elongate elements (i.e., left/right [*element1*] and left/right [*element2*]). Taking the mean length of bilateral elements was deemed reasonable following a comparison of all paired elements from CT scans of the most intact/least distorted individuals (Figure S1), however this step could also work with actual individual element lengths. The mean element lengths were then used to define the radii of two spheres, centered at the averaged positions for [*joint1*] and [*joint3*], respectively, according to their configurations established in Section 2.2.3. Based on the assumption that the distance between consecutive joints does not change (*assumption b* above), the circle formed by the intersection of the two spheres represented the solution that simultaneously met the length constraints for both elongate elements. This therefore represented the complete potential space allocation for [*joint2*] (i.e., between an arch's elongate elements), with the joint being located somewhere on the intersection circle.

The position on each arch's intersection circle closest to the averaged [joint2] coordinates was calculated and considered to be the most appropriate [joint2] location for the skeleton in “baseline” (mouth closed) position, taking anatomical constraints and error minimization into consideration. Lastly, the resultant “unilateral mean visceral arches”—with bilaterally averaged joint positions and averaged elongate element lengths—were then mirrored back across the established mid‐sagittal plane to complete the bilaterally symmetrical linear skeleton.

The bilaterally symmetrical linear skeleton (Figure 5d) acted as the map for correcting distorted specimen scans (Figure 5b). Surface geometries of cartilages were generated from segmentation label fields in Amira ZIBEdition 2021.30, using the ‘*Generate Surface’* Module (Smoothing: Constrained Smoothing, Smoothing Extent: 1), and exported as meshes in .obj format and imported into the Grasshopper plug‐in for Rhinoceros 7 (McNeel & Associates). Joint coordinates were then migrated from their scan positions to their calculated bilateral symmetry positions using Grasshopper's ‘*Orient*’ module; since each cartilage element sits between two joints, this action reorients the cartilage element mesh to reposition the segmented animal skeleton according to the joint coordinates of the corrected linear skeleton (Figure 5d).

### 2.3 Constructing a bio‐realistic and poseable parametric skeleton

The constructed symmetrical linear skeletal model (i.e., baseline skeletal positions) acted as the key input for a bio‐realistic parametric model, where the manipulation of joint position could drive cartilage movements for digital investigation of biomechanics. Parametric modeling approaches generate 3D geometry from (a) a common schema (in our case, the common topological relationships between linear skeletal members and their surface geometries), and (b) a set of variable parameters, indicating the size and placement of each element in the schema. The outcome of the model therefore changes immediately when a parameter is modified. In our model, using a similar logic to our method of skeletal deformation correction (see Section 2.2.4 above), we move joint locations in 3D space to drive a “puppet system” for the skeleton, effectively carrying the cartilage elements from baseline to destination positions (Figure 5j‐1). All elements in this kinematic chain can therefore be adjusted parametrically (i.e., joint positions and connectivity, element lengths) to account for different specimen (or even species) configurations. An additional level of parametrization comes through our conversion of the scan‐acquired skeletal element data to individual parametric models, where element geometries are not defined by their original meshes (having considerable scan resolution noise), but rather by smooth and closed spline curves that capture cross‐sectional shape. Beyond the 3D constellations of joint positions, this method renders skeletal cross‐sectional shapes both alterable and less computationally expensive, permitting further explorations of the effect of skeletal form on function (e.g., how branchial arch shape affects fluid flow into the gill pockets). For the current study, however, we focus on gross kinematic movements, where element lengths and joint positions are the relevant driving parameters.

In Grasshopper, the joints can be assigned any movement vector to enable desired kinematic movements (e.g., the change in joint positions that occur during feeding). We therefore required numerical data for joint positions at various head configurations as “destination” coordinates for each joint. These, however, are challenging to determine, due to the near impossibility of CT scanning a basking shark head with its mouth open. We therefore took advantage of several sources that offered views of basking sharks with their mouths open which, when correlated with our CT scan data, provided joint or cartilage landmarks that could be located externally. Of particular interest were the locations of elongate elements and the joints between them (see Section 2.2 above), as these undergo large excursions and therefore are likely important to the feeding mechanism (see *assumption d* above).

Firstly, a surface scan of a fresh basking shark head from Museums Victoria, suspended open‐mouthed, provided 3D anatomical information on joint and cartilage co‐movements (Figure 2 and Table S1: Shark#7). We noted that the ratio between ventral depression of the mouth and width of the oral chamber, hanging open under its own weight, was somewhat more extreme in this scan than in videos of feeding sharks (Figure 6), providing a concept of a positional extreme for arch movements (i.e., how arch positions are affected by the jaw being maximally opened). Second, videos of feeding basking sharks were acquired by our team using custom biologging tags, deployed on sharks off the Irish coast in May 2022 under license from the Health Products Regulatory Authority of Ireland (#AE19136/P127) (Shark#15). Tags were outfitted with a tethered float containing a Customized Animal Tracking Solutions (CATS) IMU tag embedded with a video camera; tags were attached to the body caudolateral to the dorsal fin, providing an anterodorsal view of feeding sharks. Underwater videos were also acquired from our workgroup from cameras mounted on tethered underwater robots (Scott et al., 2023), providing lateral views of feeding sharks. Lastly, abundant underwater videos were also available online (e.g., on YouTube; Shark#10–11), from both amateur and professional sources. For all video sources, videos were chosen where the camera's view was clear, well‐lit (i.e., where both external and internal structures of the feeding shark were visible) and stable (i.e., close to the surface, with little turbulence or suspended particulate matter) and where the animal moved slowly and consistently relative to the camera.

In addition to using videos to understand typical cranial feeding movements, video data was also used in a non‐conventional photogrammetry approach for reconstructing anatomy, to locate 3D joint coordinates as kinematic destinations for the parametric model (Figures 5f–j and 6). Photogrammetry is conventionally used to generate 3D information of an object in space, using photographs with overlapping content, taken from multiple positions around the object (Alshawabkeh et al., 2020; Guendulain‐García et al., 2023; Irschick et al., 2022; McCarthy et al., 2019; Plum & Labonte, 2021; Song et al., 2022). Given the challenge of recording basking sharks from multiple perspectives simultaneously, we instead apply what we believe is a novel approach for photogrammetry of mobile animals, deriving 3D reconstructions from videos of feeding animals (Figure 5e–h). Videos chosen were filmed with relatively static camera positions, showing sharks steadily approaching the camera and swimming past it, allowing multiple views of the same shark from considerably varied angles (i.e., creating the overlapping images needed for photogrammetry). Of the 11 videos we acquired online with reasonable quality (i.e., meeting the criteria above), two of those provided diverse enough views to allow generation of convincing 3D models of feeding basking sharks swimming close to the water surface. Frames from the two acquired videos (Figure 2 and Table S1: Sharks#10–11) were used as image sequences for photogrammetry reconstructions, processed in Agisoft Metashape Professional software, to deliver 3D surface models of feeding basking sharks. Museums Victoria surface scans (Figures 2 and 6: Shark#7) and additional videos (Shark#15) were used as references for verifying movement hypotheses (Table S1).

Photogrammetry models provided clear views of the 3D morphologies and orientations of all visceral arches in feeding positions, as well as the coordinates of key joints (see *Results/Discussion*) (Figure 5f–i). Although the arches were clearly visible, the surface of the rendered photogrammetry model was not smooth, due to noise from the reconstruction. The arches were therefore first marked by 2D curves drawn in horizontal model cross sections, from which smooth surfaces were extruded dorsoventrally. The intersections of these surfaces with the model created preliminary traces of the medial edges of the visceral arches (Figure 5g), which were then simplified in Rhino 7 by fitting smooth NURBS curves to the arch anatomy (Figure 5h). Based on our anatomical observations and CT scan data, we found [*joint1*] and [*joint3*] to be located at approximately the dorsal and ventral extremes of the internal gill slits (Figures 5h and 6). Control points for NURBS curves could therefore be used to locate joint position: the points at the ends of each arch curve indicated the positions of [*joint1*] and [*joint3*], with the point from the mid‐section of the curve acting as a first approximation of [*joint2*]'s position. Together, this constellation of joints simplified each arch to a plane describing the arch's open position (*assumption f*).

After defining joint locations for the open‐mouthed video‐photogrammetry models, the coordinate space was uniformly scaled to the size of the baseline model to allow correspondence between datasets. The scaling factor was determined by standardizing the linear distance between the left and right [*joint1*] instances of the branchial and hyoid arches, and the rostrocaudal distance between the midpoints of the paired [*joint1*] instances of the first and last branchial arches; we do not expect these distances to change during feeding, given that they capture elements embedded in muscle in the dorsal pharynx. Once models had been scaled, two operations were performed. First, the scaled open‐mouthed video‐photogrammetry model (Shark#10) and the museum‐surface‐scan model (Shark#7) were aligned as closely as possible (Figure 6); these were different individual animals but non‐feeding structures could be surprisingly well aligned (e.g., the dorsal/pectoral fin leading edges, the eyes, the dorsal body surface from chondrocranium to pharynx). This allowed comparison of arch and joint positions between a live shark actively feeding in water (Shark#10) and a dead shark with its arches hanging in air under the animal's weight (Shark#7).

Second, after aligning the baseline model with the video‐photogrammetry model, a set of linear vectors was defined for the joints in each arch to translate them from their closed (baseline) to their open (feeding) positions (Figure 5f–j). Vectors were considered to be linear travel paths, as we were primarily interested in the extremes of the system and had no positional data for arch kinematics during deployment. Based on *assumption b*, however, the trajectory of [*joint2*] will never be linear, yet the precise location of this joint is difficult to determine from video photogrammetry. We therefore used each arch's “open position plane” to constrain localization of the joint, as the plane of each arch is readily visible in photogrammetry reconstructions. In a method similar to that described in Section 2.2.4 above, two circles were drawn on each arch plane with radii equal to arch elongate element length, with the intersection points of the paired circles used as the most suitable location for [*joint2*] in the open‐mouthed position (Figure 5i). In video observations of wild sharks, expansion of the pharynx in feeding is apparently driven more by ventral depression of the mouth and throat, rather than dorsal elevation of the head and vertebral column (e.g., Figure 5f). We therefore considered the pharyngobranchial‐vertebral joint ([*joint0*]; Figure 3) to be immobile during feeding (*assumption e*).

## 3 RESULTS & DISCUSSION

Our study, combining approaches from anatomy and design fields, provides novel insights into basking shark anatomy and kinematics, but also modeling tools for challenging study species and specimens with partial and/or multi‐modal data. This integrated approach allowed us to take advantage of modern imaging, 3D reconstruction, and filming techniques for observations of basking shark functional anatomy that would have been impossible for huge aquatic animals decades ago. The spatial accuracy of our results, however, is clearly conditional on the quality of feeding videos and anatomical data. Our approach would therefore likely be less effective for many other large shark species, which have their visceral arch anatomy less visible externally; may inhabit deeper poorly lit waters; may move too quickly to allow stabilized videos of particular actions; or have poorly mineralized skeletons that cannot be resolved by CT scanning. For such challenging species, however, our models still provide an alterable baseline scaffold for investigating visceral arch mechanics and testing evolutionary and functional hypotheses.

### 3.1 Anatomical observations

**FIGURE 8:**
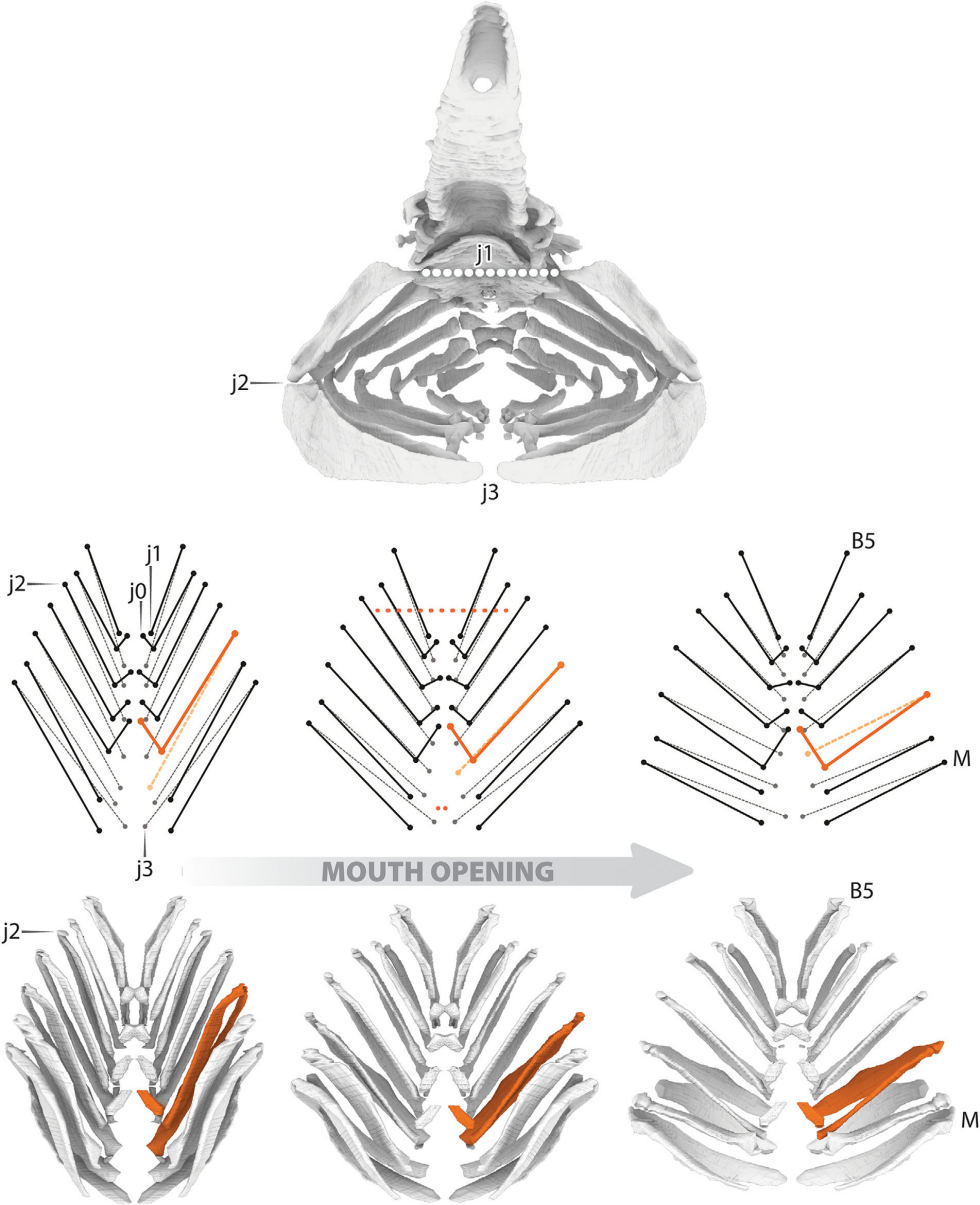
Parametric basking shark skeleton posed in half‐open configuration (anterior view, top image), illustrating the sequential alignment of arch lateral joints (*j2*) in successive visceral arches. Note also the extremely broad symphyseal joint (*j1*) between upper jaw halves in the mandibular arch, compared to the narrow ventral joint (*j3*) between lower jaw halves. The linear skeleton model (middle image row) is the basis of the “digital marionette”, where joints can be moved to mimic observed feeding positions. Once posed, the linear skeleton acts as a guide for replacing the meshed cartilages, constructing the bio‐realistic skeleton (bottom image row). The models in the bottom rows, shown in dorsal views, actuate from closed‐mouth, to half‐open, to fully‐open configurations (from left to right). The first branchial arch, highlighted in orange, illustrates how arches flare, like car doors opening. Note that flaring is less pronounced in posterior arches, with little if any movement of the fifth branchial arch (*B5*).

Despite long‐standing scientific and public interest in the basking shark and its ecology, study of its cranial anatomy has barely advanced since the first published anatomical drawings 150 years ago. Pavesi (1874) beautifully rendered visceral arch arrangements in an open‐mouthed (and apparently defleshed, but not skeletonized) animal in lateral view (Pavesi's Tav. II, Figure 5; see also our Figure 3). Pavesi's later work (1878) provided more stylized skeletal drawings of the individual arch elements articulating, but in resting configuration and from different anatomical perspectives than his previous work (Pavesi's figures 8–10; arrangements revisited and illustrated by Senna, 1925). These careful illustrations have been invaluable for determining the general size and shape of skeletal elements in basking sharks and placing them in context with other species (e.g., Compagno, 1990). However, the selective focus of the illustrations and their limited anatomical perspectives have made it difficult to interpret even basic anatomical arrangements of different structures (e.g., where gill rakers and gills are positioned relative to each other and to the gill slits) and especially how these features might reorient during feeding. As far as we know, sketches by Compagno (1990)—overlaying rough jaw arch outlines on a drawing of a feeding shark's body—and hypotheses of gill arch movement by Hallacher (1977) represent the only attempts to understand how the basking shark's skeleton moves during the notable expansion and collapse of its oropharynx.

Our renderings therefore provide the first 3D visualizations of all basking shark cranial skeletal elements in connection (e.g., Figures 3 and 4) and how these interact and reposition during feeding (Figures 6, 7, 8) (see also Paskin et al., 2022). By integrating information on both outer (soft) and inner (skeletal) morphologies from multiple specimens (Figures 1 and 2), we overcome the limitations of distorted or partial specimens and/or individual techniques. As a result, we clarify fundamental aspects of this species' anatomy, rudimentary features described decades prior for related smaller species, but never verified for basking sharks. For example, compared to CT slices previously published for other shark species (e.g., Crawford, 2014; Kamminga et al., 2017; Mollen et al., 2012), we observed that the cartilage of the basking shark's cranial skeleton is relatively poorly mineralized (Figure 1). This is in keeping with anecdotal observations of the poorly mineralized skeletons of other large and coldwater shark species, even from other lineages (Dean et al., 2015). We also observed consistent intraindividual variation in the degree of mineralization, with the arch elongate elements having a more prominent mineralized cortex than smaller dorsal elements (pharyngobranchial) and ventral elements (hypobranchials, basibranchials, basihyal). The higher radiodensity of elongate element margins allowed them to be more readily segmented (Figures 1, 3, 4, 5, 7 and 8), however, we note that the elements' articular ends tended to be less mineralized, challenging precise determination of joint morphologies from CT data.

Our multimodal imaging observations revealed several aspects of visceral arch anatomy with relevance to feeding biomechanics; we briefly note several of these, proceeding roughly in a rostrocaudal direction. The within‐element variation in mineralization we observed in elongate elements was particularly evident in the jaw arch, where the symphyseal ends of the upper jaws (palatoquadrates) were in some cases nearly indeterminable in CT scans (Figures 1, 4, 7 and 8). Despite the symphysis joint being wide and unfused (Figures 7 and 8) and the upper jaw bearing no skeletal connection to the chondrocranium, it was closely apposed to the braincase, bound in soft tissue and apparently non‐protrusible (Dean et al., 2005; Huber et al., 2019; Wilga et al., 2007). Similarly, the hyoid was tightly linked by soft tissue with the jaw arch (Figure 7), suggesting a further limitation to jaw protrusion. The jaw and hyoid arches were nearly indistinguishable in videos of feeding animals and appeared to move together as the mouth opened (Figures 6 and 7). Similarly, our video observations suggest that the basibranchial series functions as a unit, depressing smoothly during feeding (Figures 6 and 7). The poor radiodensity of the basibranchials restricted us from segmenting individual elements (Figures 7 and 8) and from determining the accuracy of Pavesi (1878) and Senna's (1925) branchial series illustrations (which differ in some key structural respects). The apparent non‐robustness of the basibranchials also suggests this may be a more flexible region of the branchial basket, which could support pharyngeal expansion during feeding.

We demonstrate that the internal gill slits (which communicate via the gill pockets to the external gill slits) are positioned anterior to their respective visceral arches (e.g., gill slit 1 is anterior to branchial arch 1; Figure 3), a fact somewhat confused in other sources (e.g., Wegner, 2015: figure 3.14). Although the resolution of our CT scans (<1 mm) did not allow for resolving individual filaments of gill rakers, we discovered that the arrays of raker filaments were sometimes visible as sheets in our medical CT datasets (Figure 4). More often, however, even when raker filaments weren't resolvable, the arrays of raker hooks were visible as continuous spline curves tracking the shape of the relevant visceral arches (e.g., Figures 1, 2, and 4). These indications of gill raker presence allowed ready determination of the locations of filtering elements. From these renderings, it could be verified that gill rakers line the aboral edges of branchial arches 2–4 (i.e., the edge furthest from the pharynx) on both rostral and caudal faces of the arches, but only the caudal face of the hyoid arch and the rostral face of branchial arch 5 (which both lack gill slits on their non‐adorned sides; Figure 4). The result is 10 complete gill raker arrays per side of the head, lining the entrances of all gill pockets, continuously flanking the rostral and caudal faces of the associated elongate elements (Figure 4). This finding supports the earliest renderings of Pavesi (1874; see our Figure 3) rather than his later (1878) renderings which suggested an interruption in the raker array at the articulation between elongate elements (e.g., *j2* in Figure 4). We also note that the gill raker arrays are always combined with gill hemibranchs (sheets of gill material on one face of the gill arch; Wegner, 2015), except for the 5th branchial arch, which lacks gill material. At the caudal end of the branchial basket, we observe no skeletal connection between branchial arch 5 and the pectoral girdle, supporting the illustrations in Senna (1925), whereas Pavesi (1874) shows them connected. This suggests freedom of movement of the branchial basket relative to the pectoral fins (e.g., the ability for the pharynx to accordion and extend even further rostrally), although our comparisons of closed‐ vs. open‐mouthed animals indicate branchial arch 5 is far less mobile than the others during feeding (Figure 8; see below).

### 3.2 Integrated techniques

The combination of CT and photogrammetry approaches allowed us to capitalize on the respective advantages of these non‐destructive techniques. Whereas CT‐scanning allows for non‐invasive visualization and rendering of an animal's internal skeleton (e.g., Figures 1, 3 and 4), photogrammetry permits integration of 2D images of a body back into 3D for specimens unsuitable for CT scanning due to their size and/or materials (e.g., Shark#6's metal support structures) (Christiansen et al., 2019; Irschick et al., 2022; Shero et al., 2021).

Compared to 3D laser scanning, which produces detailed surface geometry data, yet for relatively smaller objects and requires more expensive and specialized devices, photogrammetry better accommodates larger spatial scales and various climates and site situations (Guendulain‐García et al., 2023), making it the more logical technology for our project. Photogrammetry is widely used in studies that require imaging of large‐scale objects, contributing to satellite imagery of landscapes (Pearse et al., 2018), heritage conservation (Rocha et al., 2020) and archeology (Alshawabkeh et al., 2020; van Riel, 2024). The fields of underwater archeology and geology have explored photogrammetry particularly extensively as a tool for excavating wrecked vessels (e.g., Helfman et al., 2024) or seafloor structures, usually by applying mosaic‐like grid systems for imaging and stitching together a large ROI by aligning landmarks (Song et al., 2022).

Application of photogrammetry in the life sciences has become more common in recent years, for capturing the morphologies of body parts (e.g., Roscian et al., 2021), individual animals (e.g., Christiansen et al., 2019; Irschick et al., 2022; Plum & Labonte, 2021) or colonies of sessile organisms (Chen & Dai, 2021; Chirayath & Earle, 2016; Chirayath & Li, 2019). Photogrammetry in most of these cases, however, is typically applied either to organisms/structures small enough to bring into the lab environment or those that stay still in the wild. Photogrammetry of mobile living animals has remained challenging, most successfully relying on a network of cameras recording simultaneously from multiple angles to avoid the artifacts of animal movement (Irschick et al., 2022). Alternatively, multiple reference images of a species in the wild could be used to “sculpt” a digital surface model (“soft modeling” in animation parlance), either of a specific or average individual in a particular body position, although this requires a diversity of images of the behavior of interest and somewhat more artistic license (Segre et al., 2023).

Our method of single‐video photogrammetry is a less equipment‐intensive solution than the multi‐camera version, but avoids the challenge of keeping aquatic subjects stationary (e.g., by swimming them in flumes or taking many images quickly). Instead, we solve the obstacle of imaging the shark from diverse angles by letting the active animal do the work, deriving reconstructions from videos of the moving fish recorded by a stationary camera (Figure 5e,f), an approach more suited to our large oceanic species. Basking shark feeding is also a particularly apt study behavior for this approach, as the animals tend to maintain maximum gape for long periods (Sims, 2008) and their slow feeding nature provides gentle camera angle transitions for successful photogrammetric reconstructions from a living and moving large‐scale animal.

Due to the basking shark's huge gape, we were also able to capture anatomical features even inside the mouth (e.g., Figures 5e–h, 6 and 7). More importantly to our feeding reconstructions, the superficial positions of visceral arch elongate elements meant that relevant cartilages could be faithfully tracked from external views (unlike smaller elements like pharyngobranchials, hidden deeply embedded in tissue, Figures 5f, 6 and 7). As a result, rather than aligning cartilage elements separately, we were able to use the whole articulated piece‐wise linear skeletal system to guide the registration process. This is particularly valuable in observations of wild animals, when it is impossible to use markers to precisely triangulate elements of the skeleton (as with the radio‐opaque beads attached to skeletons in XROMM; Brainerd & Camp, 2019). Our approach could be used to look at different stages of an animal's movement beyond poses maintained statically, if enough data were available to show each kinematic stage from diverse perspectives (e.g., from multiple cameras recording simultaneously). Additionally, this technique can be applied to explore differences among individuals (as in our comparisons of in‐air and in‐water models), for understanding the roots of intraindividual performance variation.

Our hybrid CT‐photogrammetry models—blending skeletal specimens with wild animal feeding postures—formed the bases of our parametric models (Figure 5j), guiding the posing of digital renderings of actual skeletons to reconstruct life positions (Figures 7 and 8). In our digital puppets, points representing joints can be assigned coordinates or vector values, within the constraints of fixed skeletal element lengths and the hierarchy of connected elements (i.e., moving one joint will have an effect on others). The assignment of joint positions enables mimicking of kinematic movements (e.g., the changes in joint positions that occur during feeding), leading to bio‐realistic anatomical positioning of cartilages (Figures 7 and 8). A diversity of approaches has been used by researchers interested in understanding and measuring skeletal motion in animals, living and extinct, *in* and ex vivo, with the level of inference tightly linked to how amenable the species is for controlled observation and/or for invasive methods for marking and tracking skeletal elements hidden from the eye (e.g., Brainerd et al., 2010; Gatesy et al., 2010; Manafzadeh, 2020; Nyakatura et al., 2019; Westneat, 2005). The current gold standard in animal biomechanics is XROMM, which uses CT scans and fiducial markers to precisely register subject‐specific 3D skeletal data in X‐ray fluoroscopic videos (Brainerd et al., 2010; Brainerd & Camp, 2019; Manafzadeh, 2020; van Meer et al., 2019).

Our approach—acknowledging the impossibility of basking sharks in laboratory conditions—is more similar to scientific rotoscoping (Gatesy et al., 2010), a markerless method of motion analysis, where articulated skeletal models are registered with their fluoroscopic shadows in a calibrated virtual environment. Like scientific rotoscoping, our method relies largely on “forward kinematics”, employing explicit control of each joint's position to align the skeleton in a mimicry of feeding poses (Gatesy et al., 2010). We also, however, use “inverse kinematics”, to locate [*joint2*]'s position based on the geometric constraints of the skeleton; the result returned two solutions but only one was biologically realistic. This integrated approach allowed us to solve the challenge of pinpointing “hidden joints” in wild sharks and the infeasibility of observing basking sharks with X‐ray video. Currently, however, our model ignores long‐axis rotations of skeletal elements, which could be considerable (Scott et al., 2019) and important for understanding the mechanics of the visceral arches. Better understanding of how external landmarks in sharks correspond to internal skeletal features would be particularly valuable for these future pursuits (having already proven useful in study of the extreme jaw protrusion of the rarely‐observed goblin shark; Yano et al., 2007).

A major advantage of parametric models like ours for biomechanics studies is that they establish a framework that is both poseable and quantifiable. For example, by digitally wrapping a 3D convex hull around the constellation of joint coordinates in different feeding positions, we can calculate that the basking shark pharynx expands by ~450% when it opens its mouth to feed. Volume estimations in both closed and open states are surely somewhat overestimated in these calculations, given that constraints imposed by surrounding tissues on space and range of motion are not included; however, a major boon of parametric models is that they can also be iteratively refined. For example, our baseline model can be improved with every additional piece of species information, in the precision of defined joint positions, but also through entirely replacing skeletal meshes (e.g., if higher resolution scans become available, of whole heads or even individual elements). Similarly, our model allows inference of skeletal geometry for specimens with missing or damaged elements, allowing the construction of animal models even from partial datasets (commonplace in studies of large animals and/or where samples are garnered opportunistically).

Our parametric model can be easily adjusted to explore real or hypothesized individual variation and any associated measurable effects on biomechanics, allowing also for quantifiable testing of performance hypotheses (e.g., how skeletal element lengths affect mouth volume). Currently, we use the combination of anatomical and behavioral data to frame (and rule out) kinematic model possibilities for the maximum gape feeding position. For example, CT‐derived anatomy alone might suggest that all arch joints are equally mobile, but videos of open‐mouthed animals help to disprove that idea (e.g., with regard to movement of the upper jaw symphysis; Figures 7 and 8). In the future, our approach could be applied to investigate other behaviors/cranial positions, helping to better define degrees of freedom for specific joints, a mechanical challenge nearly impossible to test by other means with an animal of this size. Once established, specific joint degrees of freedom (real or hypothesized) can be incorporated into our models to determine which poses are allowable, permitting the full integration of skeletal anatomy into feeding motion analysis.

In the fields of design and architecture, from which our parametric approach was derived, parametric models are constructed to represent essential relationships of structure or geometry, while permitting specific features to vary (as parameters, e.g., floor heights in a building). Such parameterization allows design decision‐making to be deferred to a later stage or to be optimized for specific performance objectives (Aish & Woodbury, 2005; Frazer, 2016). In our study's context, we intended to first determine relevant dimensions of geometrical variance among samples, then represent each of these as defined parameters to map observed (or possible) basking shark geometries. Yet, our comparison of the ratios of elongate elements across our CT‐scanned specimens (Figure S1) and the strong alignment of in‐water and in‐air photogrammetry models (Figure 6) argued that gross skeletal proportions in the branchial basket do not change appreciably across our basking shark specimens (although overall head length has been observed to grow with negative allometry; Ahnelt et al., 2020). This suggested that a single model was adequate initially for capturing oropharynx biomechanics. In the future, the parametric model could be used to more deeply explore interindividual variability by locating particular specimens in relation to one another in order to survey the geometric range of the species. In this way, models and/or joint hierarchies can also be repurposed to reflect different individuals and species (e.g., allowing testing of branchial basket performance among different sharks). Comparison of arch proportions and symphyseal mobility among lamniform sharks, for example, would be useful for understanding which aspects of visceral arch anatomy were adapted through evolution in support of the filter‐feeding niche. With available databanks of CT data (e.g., Anon, 2025; Kamminga et al., 2017) and the advent of motion prediction neural networks (Irschick et al., 2022), our parametric model could act as a flexible baseline rigging for exploring cranial skeletal mechanics on evolutionary scales.

### 3.3 Kinematic observations

Although there are numerous scientific observations of basking shark feeding (e.g., Sims, 1999; Sims, 2008; Sims & Merrett, 1997), none to our knowledge have related aspects of cranial motion to the anatomy and arrangements of the underlying skeleton, except for brief hypotheses put forth by Hallacher (1977) and Compagno (1990). We therefore provide a concise basic description of feeding behavior derived from our video and modeling observations and with a focus on the visceral arches; a detailed quantitative description of feeding kinematics (e.g., timings of movements) will follow in another study. There is a rich literature focused on the feeding biomechanics of elasmobranch fishes, but most studies of skeletal motion have focused on movements of the jaws and hyoid in relation to feeding (e.g., Scott et al., 2019; Wilga & Sanford, 2008; Yano et al., 2007; but see van Meer et al., 2019; Goto et al., 2013).

In basking sharks, when the mouth opens as feeding bouts begin, the more caudal arches mimic this action by opening dorsoventrally (increasing the angle between elements at [*joint2*]) and depressing the ventral floor of the pharynx (i.e., [*joint3*]) (Figures 6 and 8). Although the rostrum lifts to some degree, most of the increase in pharynx height is by ventral depression (Figure 5f,j); dorsal pharyngeal elements therefore do not seem to contribute appreciably to feeding. The upper jaw does not protrude away from the chondrocranium (Figures 6 and 7), as in more macrocarnivorous lamniform sharks (Dean et al., 2005; Huber et al., 2019; Wilga, 2005; Yano et al., 2007). Our anatomical analyses imply the upper jaw may in fact be incapable of protrusion (see above), but perhaps the “fixation” of the upper jaw symphysis relative to the chondrocranium is also a necessary reinforcement against the gross reorientation and expansion of the branchial basket. As the mouth opens, all arches flare rostrally (like car doors opening), translating [*joint2*] forward, creating space between successive arches (Figures 6, 7, 8). From our CT datasets, we demonstrate that elongate elements in more rostral visceral arches are significantly longer (Figures 4 and S1). This variation allows the more rostral arches to create more vertical distance as they open, creating a conical oropharynx that tapers dorso‐caudally toward the esophagus (Figures 5e–h and 6, 7, 8). The truncated cone morphology of the throat is similar to the morphologies often used in suction feeding simulations in fishes (Day et al., 2015; Provini & van Wassenbergh, 2018). Our first volumetric visualizations of the basking shark oral cavity can also therefore offer powerful baselines for understanding and modeling filtering performance in these sharks.

Our registration of two open‐mouthed 3D models, one in air (Shark#7) and one in water (Shark#10), further demonstrates the integrated movements of branchial basket elements (Figure 6). The models overlap surprisingly well (despite being different animals in different media), but the shape of the deployed branchial basket differs between the models. The in‐air model shows a gape that is narrower but deeper, with the lateral joints of the branchial basket (i.e., [*joint2*]) folded back (positioned more medially and caudally) and the ventral joints (i.e., [*joint3*]) more ventrally depressed (Figure 6). In contrast, the in‐water model's gape is wider but less ventrally depressed, with lateral branchial basket joints more laterally and rostrally flared and ventral joints less depressed (Figure 6). This supports our modeling approach in demonstrating that the visceral arches do behave as elements of fixed lengths linked by joints. In such a kinematic articular chain, movement of any one element or joint has a cascading effect on connected elements and affiliated joints (Gatesy et al., 2010). As a result, the dropping of the jaw under its own weight in air pulls all ventral visceral arch joints downward, causing concomitant narrowing of the oral cavity (medial movement of lateral joints).

Obviously, the in‐air model does not completely collapse medially (i.e., achieve the full extent of medial movement imaginable from element lengths and joint positions). This illustrates that soft tissues and perhaps joint morphologies also control and limit arch movements (Huber et al., 2019; Wilga et al., 2007). Future models should incorporate these important supporting materials; currently, however, no information exists, for example, on the properties of basking shark skin or the location and function of their tendons with regard to arch movements. It is worth noting that in the living animal (in‐water model), the arches are more “flared” in the rostral direction (Figure 6). This important movement—ensuring adequate distance between arches during filtration (Figure 8)—is therefore likely not just a passive result of opening the mouth and depressing the arches (unless the weight of the ventral body ‘overrides’ that). Articulated biophysical models, incorporating the cross‐sectional shapes of the elongate elements, would be useful for determining the degree to which flow through the mouth and out the gill slits is responsible for passively erecting the arches in this way.

## AUTHOR CONTRIBUTIONS

**Tairan Li:** Conceptualization; investigation; writing – original draft; writing – review and editing; visualization; validation; methodology; software; formal analysis; data curation. **Mike Schindler:** Investigation; writing – original draft; writing – review and editing; visualization; validation; formal analysis. **Martha Paskin:** Conceptualization; investigation; methodology; writing – review and editing; software; formal analysis. **Venkata A. Surapaneni:** Investigation; writing – review and editing; formal analysis; supervision; resources. **Elliott Scott:** Data curation; resources; software; formal analysis; methodology; visualization; writing – review and editing; investigation. **Sabine Hauert:** Investigation; funding acquisition; writing – review and editing; methodology; visualization; project administration; resources; supervision. **Nicholas Payne:** Investigation; conceptualization; funding acquisition; validation; visualization; writing – review and editing; project administration; resources; supervision. **David E. Cade:** Investigation; methodology; visualization; writing – review and editing; resources; data curation. **Jeremy A. Goldbogen:** Investigation; funding acquisition; methodology; visualization; writing – review and editing; supervision; resources; project administration; data curation. **Frederik H. Mollen:** Writing – original draft; investigation; conceptualization; methodology; validation; visualization; writing – review and editing; formal analysis; resources; supervision; data curation. **Daniel Baum:** Conceptualization; investigation; methodology; visualization; writing – review and editing; software; resources; supervision. **Sean Hanna:** Conceptualization; investigation; funding acquisition; writing – review and editing; methodology; software; project administration; supervision; resources; writing – original draft; formal analysis. **Mason N. Dean:** Investigation; funding acquisition; writing – original draft; conceptualization; methodology; validation; visualization; writing – review and editing; project administration; formal analysis; data curation; supervision; resources.

## FUNDING INFORMATION

The Human Frontier Science Program (HFSP), Award number: RGP0010‐2020.

## Supporting information


**FIGURE S1:** Comparison of elongate elements across three basking sharks of different sizes. The panel shows surface renderings of CT scans of *element1* (left) and *element2* (right), for all visceral arches, moving rostrocaudally, from the mandibular arch to branchial arch #5, from the bottom to the top of the figure. Left and right elements are shown for Shark#1, #2 and #4 in columns (visual color‐coded key for specimens at top of image).

**FIGURE S2:** Despite variation in animal size, elements have a similar shape. When length of elements is scaled in the graphs relative to branchial basket length (a proxy for body size, not expected to change with mouth movement and distortion of specimens; Figure 8), relative element length is similar across specimens (note the consistent slopes). Since relative element length (i.e., the distance between joints) is key for the biomechanics of our models (Figures 5 and 8), this indicates that our determined model architecture is applicable across specimens. Note the increase in relative arch element length in more rostral arches, which contributes to the conical shape of the pharynx, once open and expanded.

**TABLE S1:** List of specimens used in the current study; see Figures 1 and 2 in the main text for images of each. Information on specimens and their analyses are provided, including notes on specimen condition.

## References

[ar25693-bib-0001] Ahnelt, H. , Sauberer, M. , Ramler, D. , Koch, L. , & Pogoreutz, C. (2020). Negative allometric growth during ontogeny in the large pelagic filter‐feeding basking shark. Zoomorphology, 139(1), 71–83.

[ar25693-bib-0002] Aish, R. , & Woodbury, R. (2005). Multi‐level interaction in parametric design. Lecture Notes in Computer Science (Vol. 3638, pp. 151–162). Springer Berlin Heidelberg.

[ar25693-bib-0003] Alshawabkeh, Y. , el‐Khalili, M. , Almasri, E. , Bala'awi, F. , & al‐Massarweh, A. (2020). Heritage documentation using laser scanner and photogrammetry. The case study of Qasr Al‐Abidit, Jordan. Digital Applications in Archaeology and Cultural Heritage, 16, e00133.

[ar25693-bib-0004] Anon . (2025). Chondrichthyan Tree of Life. https://sharksrays.org/

[ar25693-bib-0005] Brainerd, E. L. , Baier, D. B. , Gatesy, S. M. , Hedrick, T. L. , Metzger, K. A. , Gilbert, S. L. , & Crisco, J. J. (2010). X‐ray reconstruction of moving morphology (XROMM): Precision, accuracy and applications in comparative biomechanics research. Journal of Experimental Zoology. Part A, Ecological Genetics and Physiology, 313(5), 262–279. 10.1002/jez.589 20095029

[ar25693-bib-0006] Brainerd, E. L. , & Camp, A. L. (2019). Functional morphology of vertebrate feeding systems: New insights from XROMM and fluoromicrometry. In V. Bels & I. Q. Whishaw (Eds.), Feeding in vertebrates: Evolution, morphology, behavior, biomechanics (pp. 21–44). Springer International Publishing.

[ar25693-bib-0007] Cade, D. E. , Levenson, J. J. , Cooper, R. , de la Parra, R. , Webb, D. H. , & Dove, A. D. M. (2020). Whale sharks increase swimming effort while filter feeding, but appear to maintain high foraging efficiencies. The Journal of Experimental Biology, 223(11), jeb224402. 10.1242/jeb.224402 32366692

[ar25693-bib-0008] Carey, N. , & Goldbogen, J. A. (2017). Kinematics of ram filter feeding and beat‐glide swimming in the northern anchovy *Engraulis mordax* . The Journal of Experimental Biology, 220(15), 2717–2725. 28495869 10.1242/jeb.158337

[ar25693-bib-0009] Chen, G. K. , & Dai, C. F. (2021). Using 3D photogrammetry to quantify the subtle differences of coral reefs under the impacts of marine activities. Marine Pollution Bulletin, 173, 113032. 34689075 10.1016/j.marpolbul.2021.113032

[ar25693-bib-0010] Chirayath, V. , & Earle, S. A. (2016). Drones that see through waves: Preliminary results from airborne fluid lensing for centimetre‐scale aquatic conservation. Aquatic Conservation: Marine and Freshwater Ecosystems, 26, 237–250.

[ar25693-bib-0011] Chirayath, V. , & Li, A. (2019). Next‐generation optical sensing technologies for exploring ocean worlds: NASA FluidCam, MiDAR, and NeMO‐Net. Frontiers in Marine Science, 6, 521.

[ar25693-bib-0012] Christiansen, F. , Sironi, M. , Moore, M. J. , di Martino, M. , Ricciardi, M. , Warick, H. A. , Irschick, D. J. , Gutierrez, R. , & Uhart, M. M. (2019). Estimating body mass of free‐living whales using aerial photogrammetry and 3D volumetrics. Methods in Ecology and Evolution, 10(12), 2034–2044.

[ar25693-bib-0013] Cione, A. L. , & Reguero, M. A. (1998). A middle Eocene basking shark (Lamniformes, Cetorhinidae) from Antarctica. Antarctic Science, 10(1), 83–88.

[ar25693-bib-0014] Compagno, L. (1990). Relationships of the megamouth shark, *Megachasma pelagios* (Lamniformes, Megachasmidae), with comments on its feeding habits. NOAA technical report NMFS. https://www.vliz.be/imisdocs/publications/ocrd/39492.pdf

[ar25693-bib-0015] Crawford, C. (2014). Skeletal Anatomy in the Chondrichthyan Tree of Life. Ph.D. in Marine Biology. College of Charleston.

[ar25693-bib-0016] da Cunha Ramos, H. , Gomes, H. , Colosio, A. C. , Marcondes, M. C. C. , Lopez, R. P. G. , Michalski, B. E. , Ghisolfi, R. D. , Gonçalves, M. I. C. , & Bovendorp, R. S. (2024). Postmortem interval applied to cetacean carcasses: Observations from laboratory and field studies with the Abrolhos Bank region, Brazil. Forensic Science International: Animals and Environments, 5, 100082.

[ar25693-bib-0096] de Carvalho, M. (1996). Higher‐level elasmobranch phylogeny, basal Squaleans, and paraphyly. In M. L. J. Stiassny, L. R. Parenti & G. D. Johnson (Eds.), Interrelationships of fishes (pp. 35–62). Academic Press.

[ar25693-bib-0017] Day, S. W. , Higham, T. E. , Holzman, R. , & van Wassenbergh, S. (2015). Morphology, kinematics, and dynamics: The mechanics of suction feeding in fishes. Integrative and Comparative Biology, 55(1), 21–35. 25980568 10.1093/icb/icv032

[ar25693-bib-0018] Dean, M. N. , Ekstrom, L. , Monsonego‐Ornan, E. , Ballantyne, J. , Witten, P. E. , Riley, C. , Habraken, W. , & Omelon, S. (2015). Mineral homeostasis and regulation of mineralization processes in the skeletons of sharks, rays and relatives (Elasmobranchii). Seminars in Cell & Developmental Biology, 46, 51–67. 26546857 10.1016/j.semcdb.2015.10.022

[ar25693-bib-0019] Dean, M. N. , Wilga, C. D. , & Summers, A. P. (2005). Eating without hands or tongue: Specialization, elaboration and the evolution of prey processing mechanisms in cartilaginous fishes. Biology Letters, 1(3), 357–361. 17148206 10.1098/rsbl.2005.0319 PMC1617152

[ar25693-bib-0020] Divi, R. V. , Strother, J. A. , & Misty Paig‐Tran, E. W. (2018). Manta rays feed using ricochet separation, a novel nonclogging filtration mechanism. Science Advances, 4(9), eaat9533. 30263959 10.1126/sciadv.aat9533 PMC6157963

[ar25693-bib-0021] Dolton, H. R. , Jackson, A. L. , Deaville, R. , Hall, J. , Hall, G. , McManus, G. , Perkins, M. W. , Rolfe, R. A. , Snelling, E. P. , Houghton, J. D. R. , Sims, D. W. , & Payne, N. L. (2023). Regionally endothermic traits in planktivorous basking sharks *Cetorhinus maximus* . Endangered Species Research, 51, 227–232.

[ar25693-bib-0022] Fahlman, A. , Aoki, K. , Bale, G. , Brijs, J. , Chon, K. H. , Drummond, C. K. , Føre, M. , Manteca, X. , McDonald, B. I. , McKnight, J. C. , Sakamoto, K. Q. , Suzuki, I. , Rivero, M. J. , Ropert‐Coudert, Y. , & Wisniewska, D. M. (2021). The new era of physio‐logging and their grand challenges. Frontiers in Physiology, 12, 669158. 33859577 10.3389/fphys.2021.669158 PMC8042203

[ar25693-bib-0023] Frazer, J. (2016). Parametric computation: History and future. Architectural Design, 86(2), 18–23.

[ar25693-bib-0024] Friedman, M. (2012). Parallel evolutionary trajectories underlie the origin of giant suspension‐feeding whales and bony fishes. Proceedings of the Royal Society B: Biological Sciences, 279(1730), 944–951. 10.1098/rspb.2011.1381 PMC3259929 21849314

[ar25693-bib-0025] Friedman, M. , Shimada, K. , Martin, L. D. , Everhart, M. J. , Liston, J. , Maltese, A. , & Triebold, M. (2010). 100‐million‐year dynasty of giant planktivorous bony fishes in the Mesozoic seas. Science, 327(5968), 990–993. 20167784 10.1126/science.1184743

[ar25693-bib-0026] Fudge, D. S. , Szewciw, L. J. , & Schwalb, A. N. (2009). Morphology and development of blue whale baleen: An annotated translation of Tycho tullberg's classic 1883 paper. Aquatic Mammals, 35(2), 226–252.

[ar25693-bib-0027] Gatesy, S. M. , Baier, D. B. , Jenkins, F. A. , & Dial, K. P. (2010). Scientific rotoscoping: A morphology‐based method of 3‐D motion analysis and visualization. Journal of Experimental Zoology. Part A, Ecological Genetics and Physiology, 313(5), 244–261. 20084664 10.1002/jez.588

[ar25693-bib-0028] Goldbogen, J. A. , Cade, D. E. , Boersma, A. T. , Calambokidis, J. , Kahane‐Rapport, S. R. , Segre, P. S. , Stimpert, A. K. , & Friedlaender, A. S. (2017). Using digital tags with integrated video and inertial sensors to study moving morphology and associated function in large aquatic vertebrates. Anatomical Record, 300(11), 1935–1941. 10.1002/ar.23650 28971623

[ar25693-bib-0029] Goldbogen, J. A. , Cade, D. E. , Calambokidis, J. , Friedlaender, A. S. , Potvin, J. , Segre, P. S. , & Werth, A. J. (2017). How baleen whales feed: The biomechanics of engulfment and filtration. Annual Review of Marine Science, 9, 367–386. 10.1146/annurev-marine-122414-033905 27620830

[ar25693-bib-0030] Gore, M. , Camplisson, E. , & Ormond, R. (2023). The biology and ecology of the basking shark: A review. Advances in Marine Biology, 95, 113–257. 37923538 10.1016/bs.amb.2023.08.005

[ar25693-bib-0031] Goto, T. , Shiba, Y. , Shibagaki, K. , & Nakaya, K. (2013). Morphology and ventilatory function of gills in the carpet shark family Parascylliidae (Elasmobranchii, Orectolobiformes). Zoological Science, 30(6), 461–468. 23721470 10.2108/zsj.30.461

[ar25693-bib-0032] Gross‐Lerner, H. (1957). Über Bau und Entwicklung der Reusenzähne von *Cetorhinus maximus* Gunner. Zeitschrift für Zellforschung Und Mikroskopische Anatomie, 46(3), 357–386. 13531601

[ar25693-bib-0033] Guendulain‐García, S. D. , Lopez‐Beltran, A. , Banaszak, A. T. , Álvarez‐Filip, L. , Ramírez‐Chávez, E. , García‐Medrano, D. , Sellares‐Blasco, R. , & López‐Pérez, A. (2023). Photogrammetry for coral structural complexity: What is beyond sight? Coral Reefs, 42(3), 635–644.

[ar25693-bib-0034] Gunnerus, J. E. (1765). Brugden (*Squalus maximus*). Det Kongelige Norske Videnskabers Selskabs Skrifter, 3, 33–49.

[ar25693-bib-0035] Hallacher, L. E. (1977). On the feeding behavior of the basking shark, *Cetorhinus maximus* . Environmental Biology of Fishes, 2(3), 297–298. 10.1007/BF00005996

[ar25693-bib-0036] Helfman, N. , Cohen, M. , Rott, J. , Creisher, M. , & Cvikel, D. (2024). From shipwreck to seaworthy: A digital reconstruction of the Ma'agan Mikhael B ship. Digital Applications in Archaeology and Cultural Heritage, 34, e00346.

[ar25693-bib-0037] Huber, D. , Wilga, C. , Dean, M. , Ferry, L. , Gardiner, J. , Habegger, L. , Papastamatiou, Y. , Ramsay, J. , & Whitenack, L. (2019). Feeding in cartilaginous fishes: An interdisciplinary synthesis. In V. Bels & I. Q. Whishaw (Eds.), Feeding in vertebrates: Evolution, morphology, behavior, biomechanics (pp. 231–295). Springer International Publishing.

[ar25693-bib-0038] Irschick, D. J. , Christiansen, F. , Hammerschlag, N. , Martin, J. , Madsen, P. T. , Wyneken, J. , Brooks, A. , Gleiss, A. , Fossette, S. , Siler, C. , Gamble, T. , Fish, F. , Siebert, U. , Patel, J. , Xu, Z. , Kalogerakis, E. , Medina, J. , Mukherji, A. , Mandica, M. , … Lauder, G. (2022). 3D visualization processes for recreating and studying organismal form. iScience, 25(9), 104867. 36060053 10.1016/j.isci.2022.104867 PMC9437858

[ar25693-bib-0039] Janaway, R. C. , Percival, S. L. , & Wilson, A. S. (2009). Decomposition of human remains. In S. L. Percival (Ed.), Microbiology and aging: Clinical manifestations (pp. 313–334). Humana Press.

[ar25693-bib-0040] Kamminga, P. , de Bruin, P. W. , Geleijns, J. , & Brazeau, M. D. (2017). X‐ray computed tomography library of shark anatomy and lower jaw surface models. Scientific Data, 4, 170047. 28398352 10.1038/sdata.2017.47 PMC5387928

[ar25693-bib-0041] Manafzadeh, A. R. (2020). A practical guide to measuring ex vivo joint mobility using XROMM. Integrative Organismal Biology, 2(1), obaa041. 10.1093/iob/obaa041 33791578 PMC7810577

[ar25693-bib-0042] Matthews, L. H. , & Parker, H. W. (1950). Notes on the anatomy and biology of the basking shark (*Cetorhinus maximus* (Gunner)). Proceedings of the Zoological Society of London, 120, 535–576.

[ar25693-bib-0043] McCarthy, J. , Benjamin, J. , Winton, T. , & van Duivenvoorde, W. (2019). The rise of 3D in maritime archaeology. 3D Recording and Interpretation for Maritime Archaeology, 33, 1–10.

[ar25693-bib-0044] Misty Paig‐Tran, E. W. , & Summers, A. P. (2014). Comparison of the structure and composition of the branchial filters in suspension feeding elasmobranchs. The Anatomical Record, 297(4), 701–715. 24443216 10.1002/ar.22850

[ar25693-bib-0045] Mollen, F. H. , Wintner, S. P. , Iglesias, S. P. , van Sommeran, S. , & Jagt, J. W. M. (2012). Comparative morphology of rostral cartilages in extant mackerel sharks (Chondrichthyes, Lamniformes, Lamnidae) using CT scanning. Zootaxa, 3340(1), 29–43. 10.11646/zootaxa.3340.1.2

[ar25693-bib-0046] Montero‐Quintana, A. N. , Ocampo‐Valdez, C. F. , Vázquez‐Haikin, J. A. , Sosa‐Nishizaki, O. , & Osorio‐Beristain, M. (2021). Whale shark (*Rhincodon typus*) predatory flexible feeding behaviors on schooling fish. Journal of Ethology, 39(3), 399–410.

[ar25693-bib-0047] Moore, M. J. , Mitchell, G. H. , Rowles, T. K. , & Early, G. (2020). Dead cetacean? Beach, bloat, float, sink. Frontiers in Marine Science, 7, 333.

[ar25693-bib-0048] Motta, P. J. , Maslanka, M. , Hueter, R. E. , Davis, R. L. , de la Parra, R. , Mulvany, S. L. , Habegger, M. L. , Strother, J. A. , Mara, K. R. , Gardiner, J. M. , Tyminski, J. P. , & Zeigler, L. D. (2010). Feeding anatomy, filter‐feeding rate, and diet of whale sharks *Rhincodon typus* during surface ram filter feeding off the Yucatan peninsula, Mexico. Zoology, 113(4), 199–212. 10.1016/j.zool.2009.12.001 20817493

[ar25693-bib-0049] Nakaya, K. , Matsumoto, R. , & Suda, K. (2008). Feeding strategy of the megamouth shark *Megachasma pelagios* (Lamniformes: Megachasmidae). Journal of Fish Biology, 73(1), 17–34.

[ar25693-bib-0050] Nyakatura, J. A. , Melo, K. , Horvat, T. , Karakasiliotis, K. , Allen, V. R. , Andikfar, A. , Andrada, E. , Arnold, P. , Lauströer, J. , Hutchinson, J. R. , Fischer, M. S. , & Ijspeert, A. J. (2019). Reverse‐engineering the locomotion of a stem amniote. Nature, 565(7739), 351–355. 30651613 10.1038/s41586-018-0851-2

[ar25693-bib-0051] Paig‐Tran, E. W. M. , Bizzarro, J. J. , Strother, J. A. , & Summers, A. P. (2011). Bottles as models: Predicting the effects of varying swimming speed and morphology on size selectivity and filtering efficiency in fishes. The Journal of Experimental Biology, 214(Pt 10), 1643–1654. 21525310 10.1242/jeb.048702

[ar25693-bib-0052] Paskin, M. , Baum, D. , Dean, M. N. , & von Tycowicz, C. (2022). A Kendall shape space approach to 3D shape estimation from 2D landmarks. In Computer vision: ECCV 2022 (pp. 363–379). Springer Nature Switzerland.

[ar25693-bib-0053] Pavesi, P. (1874). Contribuzione alla storia naturale del genera Selache. Annali del Museo Civico di Storia Naturale di Genova, 6, 5–72.

[ar25693-bib-0054] Pavesi, P. (1878). Seconda contribuzione alla morfologia e sistemática dei Selachi. Annali del Museo Civico di Storia Naturale di Genova, 12, 348–418.

[ar25693-bib-0055] Pearse, G. D. , Dash, J. P. , Persson, H. J. , & Watt, M. S. (2018). Comparison of high‐density LiDAR and satellite photogrammetry for forest inventory. ISPRS Journal of Photogrammetry and Remote Sensing: Official Publication of the International Society for Photogrammetry and Remote Sensing (ISPRS), 142, 257–267.

[ar25693-bib-0056] Plum, F. , & Labonte, D. (2021). *scAnt*: An open‐source platform for the creation of 3D models of arthropods (and other small objects). PeerJ, 9, e11155. 33954036 10.7717/peerj.11155 PMC8048404

[ar25693-bib-0057] Prokofiev, A. M. , & Sychevskaya, E. K. (2018). Basking shark (Lamniformes: Cetorhinidae) from the lower Oligocene of the Caucasus. Journal of Ichthyology, 58(2), 127–138.

[ar25693-bib-0058] Provini, P. , & van Wassenbergh, S. (2018). Hydrodynamic performance of suction feeding is virtually unaffected by variation in the shape of the posterior region of the pharynx in fish. Royal Society Open Science, 5(9), 181249. 30839768 10.1098/rsos.181249 PMC6170587

[ar25693-bib-0059] Rigby, C. L. , et al. (2021). *Cetorhinus maximus* (amended version of 2019 assessment). The IUCN Red List of Threatened Species, p.e.T4292A194720078.

[ar25693-bib-0060] Rocha, G. , Mateus, L. , Fernández, J. , & Ferreira, V. (2020). A scan‐to‐BIM methodology applied to heritage buildings. Heritage, 3(1), 47–67.

[ar25693-bib-0061] Roscian, M. , Herrel, A. , Cornette, R. , Delapré, A. , Cherel, Y. , & Rouget, I. (2021). Underwater photogrammetry for close‐range 3D imaging of dry‐sensitive objects: The case study of cephalopod beaks. Ecology and Evolution, 11(12), 7730–7742. 34188847 10.1002/ece3.7607 PMC8216959

[ar25693-bib-0062] Sanderson, L. , Cech, S. , & Cheer, A. (1994). Paddlefish buccal flow velocity during ram suspension feeding and ram ventilation. The Journal of Experimental Biology, 186(1), 145–156. 9317518 10.1242/jeb.186.1.145

[ar25693-bib-0063] Sanderson, S. L. , Roberts, E. , Lineburg, J. , & Brooks, H. (2016). Fish mouths as engineering structures for vortical cross‐step filtration. Nature Communications, 7, 11092. 10.1038/ncomms11092 PMC4820540 27023700

[ar25693-bib-0064] Schnakenbeck, W. (1955). Der Kiemenreusenapparat vom Riesenhai (*Cetorhinus maximus*). Zoologischer Anzeiger, 154(5–6), 99–108.

[ar25693-bib-0065] Scott, B. , Wilga, C. A. D. , & Brainerd, E. L. (2019). Skeletal kinematics of the hyoid arch in the suction‐feeding shark *Chiloscyllium plagiosum* . The Journal of Experimental Biology, 222(5), jeb193573. 30824570 10.1242/jeb.193573

[ar25693-bib-0066] Scott, E. , Cade, D. , Payne, N. , & Hauert, S. (2023). Designing a system for underwater imaging and monitoring of Basking Sharks (*Cetorhinus maximus*). Authorea Preprints. https://www.authorea.com/users/698205/articles/685988‐designing‐a‐system‐for‐underwater‐imaging‐and‐monitoring‐of‐basking‐sharks‐cetorhinus‐maximus?commit=94b527e994b6729c356eca0baf5550f2d1e44d4d

[ar25693-bib-0067] Segre, P. S. , Martin, J. , Irschick, D. J. , & Goldbogen, J. A. (2023). A three‐dimensional, dynamic blue whale model for research and scientific communication. Marine Mammal Science, 39(3), 1011–1018.

[ar25693-bib-0068] Senna, A. (1925). Contributo alla conoscenza del cranio della Selache (*Cetorhinus maximus*) Gunn. Archivio Italiano di Anatomia e di Embriologia. Italian Journal of Anatomy and Embryology, 22, 84–122.

[ar25693-bib-0069] Shero, M. R. , Dale, J. , Seymour, A. C. , Hammill, M. O. , Mosnier, A. , Mongrain, S. , & Johnston, D. W. (2021). Tracking wildlife energy dynamics with unoccupied aircraft systems and three‐dimensional photogrammetry. Methods in Ecology and Evolution, 12(12), 2458–2472.

[ar25693-bib-0070] Simon, M. , Johnson, M. , & Madsen, P. T. (2012). Keeping momentum with a mouthful of water: Behavior and kinematics of humpback whale lunge feeding. The Journal of Experimental Biology, 215(Pt 21), 3786–3798. 23053368 10.1242/jeb.071092

[ar25693-bib-0071] Simon, M. , Johnson, M. , Tyack, P. , & Madsen, P. T. (2009). Behaviour and kinematics of continuous ram filtration in bowhead whales (*Balaena mysticetus*). Proceedings of the Royal Society B: Biological Sciences, 276(1674), 3819–3828. 10.1098/rspb.2009.1135 PMC2817290 19692400

[ar25693-bib-0072] Sims, D. W. (1999). Threshold foraging behaviour of basking sharks on zooplankton: Life on an energetic knife‐edge? Proceedings of the Royal Society B: Biological Sciences, 266(1427), 1437–1443.

[ar25693-bib-0073] Sims, D. W. (2008). Sieving a living: A review of the biology, ecology and conservation status of the plankton‐feeding basking shark *Cetorhinus maximus* . In Advances in marine biology (pp. 171–220). Academic Press. 10.1016/S0065-2881(08)00003-5 18929065

[ar25693-bib-0074] Sims, D. W. , & Merrett, D. A. (1997). Determination of zooplankton characteristics in the presence of surface feeding basking sharks *Cetorhinus maximus* . Marine Ecology Progress Series, 158, 297–302.

[ar25693-bib-0075] Song, Y. , Nakath, D. , She, M. , & Köser, K. (2022). Optical imaging and image restoration techniques for deep ocean mapping: A comprehensive survey. PFG – Journal of Photogrammetry Remote Sensing and Geoinformation Science, 90(3), 243–267. 10.1007/s41064-022-00206-y

[ar25693-bib-0076] Stiefel, K. M. (2021). Evolutionary trends in large pelagic filter‐feeders. Historical Biology, 33(9), 1477–1488.

[ar25693-bib-0077] Szewciw, L. J. , de Kerckhove, D. G. , Grime, G. W. , & Fudge, D. S. (2010). Calcification provides mechanical reinforcement to whale baleen alpha‐keratin. Proceedings of the Royal Society B: Biological Sciences, 277(1694), 2597–2605. 10.1098/rspb.2010.0399 PMC2982044 20392736

[ar25693-bib-0078] Tomita, T. , Sato, K. , Suda, K. , Kawauchi, J. , & Nakaya, K. (2011). Feeding of the megamouth shark (Pisces: Lamniformes: Megachasmidae) predicted by its hyoid arch: A biomechanical approach. Journal of Morphology, 272(5), 513–524. 21381075 10.1002/jmor.10905

[ar25693-bib-0079] Turner, W. (1880). The structure of the comb‐like branchial appendages and of the teeth of the basking shark. Journal of Anatomy and Physiology, 14, 273–286. 10.1017/s0370164600044163 PMC1309948 17231325

[ar25693-bib-0080] van der Hoop, J. M. , Nousek‐McGregor, A. E. , Nowacek, D. P. , Parks, S. E. , Tyack, P. , & Madsen, P. T. (2019). Foraging rates of ram‐filtering North Atlantic right whales. Functional Ecology, 33(7), 1290–1306.

[ar25693-bib-0081] van Meer, N. M. M. E. , Weller, H. I. , Manafzadeh, A. R. , Kaczmarek, E. B. , Scott, B. , Gussekloo, S. W. S. , Wilga, C. D. , Brainerd, E. L. , & Camp, A. L. (2019). Intra‐oropharyngeal food transport and swallowing in white‐spotted bamboo sharks. The Journal of Experimental Biology, 222(22), jeb201426. 10.1242/jeb.201426 31672726

[ar25693-bib-0082] van Riel, S. (2024). Applying 3D stratigraphic reconstruction to a large rescue archaeology site. A case study of the medieval and early modern submarine topography in Oslo, Norway. Digital Applications in Archaeology and Cultural Heritage, 34, e00357.

[ar25693-bib-0097] Versluys, J. (1922). Über die Rückbildung der Kiemenbogen bie den Selachii. Bijdragen tot de Dierkunde, 22(2), 95–105.

[ar25693-bib-0083] Watanabe, Y. Y. , & Papastamatiou, Y. P. (2023). Biologging and biotelemetry: Tools for understanding the lives and environments of marine animals. Annual Review of Animal Biosciences, 11, 247–267. 36790885 10.1146/annurev-animal-050322-073657

[ar25693-bib-0084] Wegner, N. C. (2015). 3: elasmobranch gill structure. In R. E. Shadwick , A. P. Farrell , & C. J. Brauner (Eds.), Fish physiology (pp. 101–151). Academic Press.

[ar25693-bib-0085] Welton, B. J. (2013a). A new archaic basking shark (Lamniformes: Cetorhinidae) from the late Eocene of western Oregon, U.S.A., and description of the dentition, gill rakers and vertebrae of the recent basking shark *Cetorhinus maximus* (Gunnerus). New Mexico Museum of Natural History and Science Bulletin, 58, 1–48.

[ar25693-bib-0086] Welton, B. J. (2013b). *Cetorhinus cf. C. maximus* (Gunnerus) (Lamniformes: Cetorhinidae), a basking shark from the late Miocene empire formation, Coos Bay, Oregon. Bulletin: Southern California Academy of Sciences, 112(2), 74–92. 10.3160/0038-3872-112.2.74

[ar25693-bib-0087] Werth, A. J. (2004). Models of hydrodynamic flow in the bowhead whale filter feeding apparatus. The Journal of Experimental Biology, 207(20), 3569–3580. 15339953 10.1242/jeb.01202

[ar25693-bib-0088] Werth, A. J. (2013). Flow‐dependent porosity and other biomechanical properties of mysticete baleen. The Journal of Experimental Biology, 216(7), 1152–1159. 23487267 10.1242/jeb.078931

[ar25693-bib-0089] Werth, A. J. , & Potvin, J. (2016). Baleen hydrodynamics and morphology of cross‐flow filtration in balaenid whale suspension feeding. PLoS One, 11(2), e0150106. 26918630 10.1371/journal.pone.0150106 PMC4769178

[ar25693-bib-0090] Westneat, M. W. (2005). Skull biomechanics and suction feeding in fishes. In Fish physiology (pp. 29–75). Academic Press.

[ar25693-bib-0091] Wilga, C. D. (2005). Morphology and evolution of the jaw suspension in lamniform sharks. Journal of Morphology, 265(1), 102–119. 15880740 10.1002/jmor.10342

[ar25693-bib-0092] Wilga, C. D. , Motta, P. J. , & Sanford, C. P. (2007). Evolution and ecology of feeding in elasmobranchs. Integrative and Comparative Biology, 47(1), 55–69. 21672820 10.1093/icb/icm029

[ar25693-bib-0093] Wilga, C. D. , & Sanford, C. P. (2008). Suction generation in white‐spotted bamboo sharks *Chiloscyllium plagiosum* . The Journal of Experimental Biology, 211(Pt 19), 3128–3138. 10.1242/jeb.018002 18805812

[ar25693-bib-0094] Yano, K. , Miya, M. , Aizawa, M. , & Noichi, T. (2007). Some aspects of the biology of the goblin shark, *Mitsukurina owstoni*, collected from the Tokyo submarine canyon and adjacent waters, Japan. Ichthyological Research, 54(4), 388–398. 10.1007/s10228-007-0414-2

