# Supplementary figures and images for: Functional models from limited data: A parametric and multimodal approach to anatomy and 3D kinematics of feeding in basking sharks (Cetorhinus maximus)
Source: Anat Rec (Hoboken). 2025 Jun 9;309(9):2262–85. doi: 10.1002/ar.25693 (PMC13431931; doi:10.1002/ar.25693)

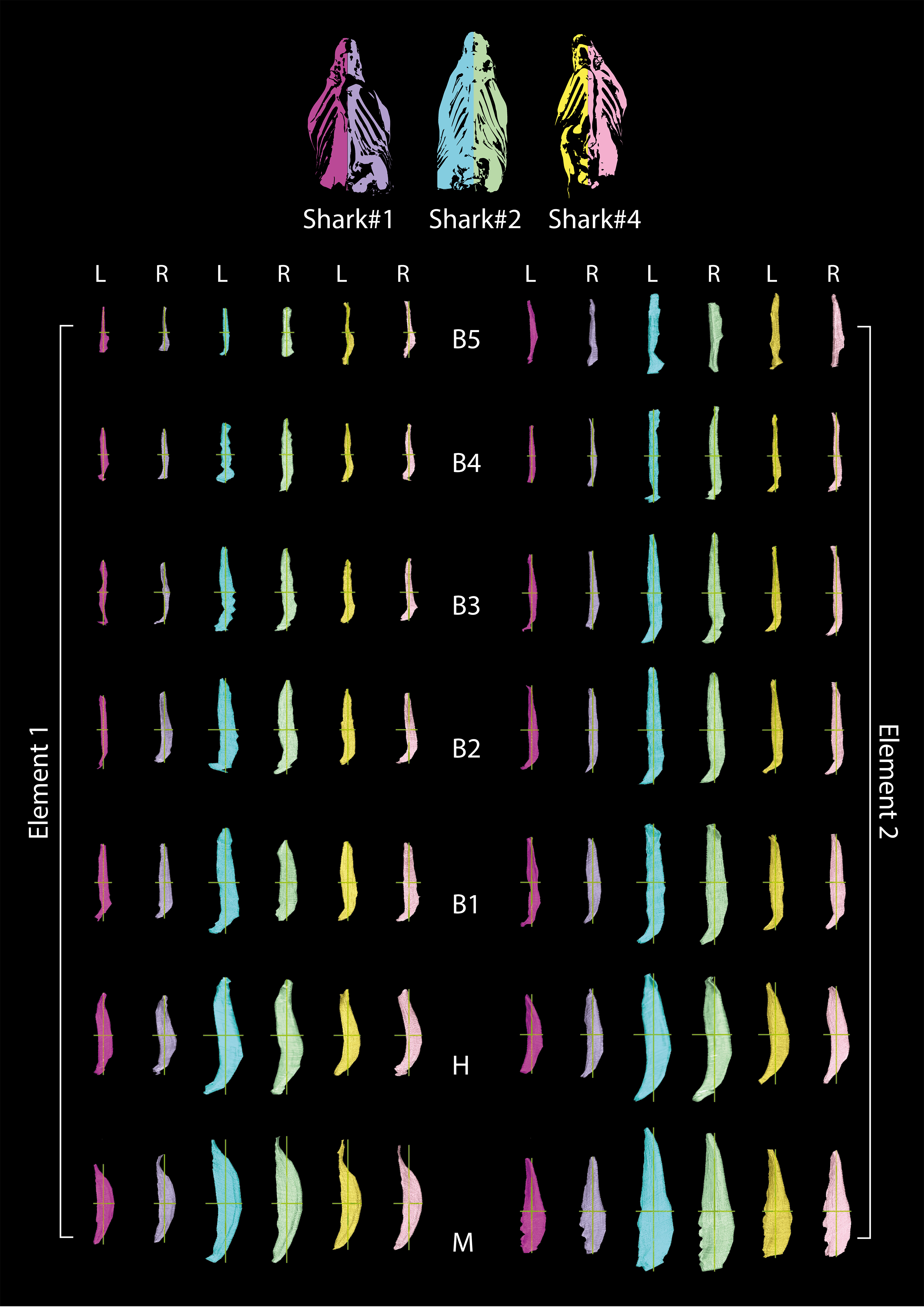

Supplement: Supplementary file 1 — FIGURE S1: Comparison of elongate elements across three basking sharks of different sizes. The panel shows surface renderings of CT scans of element1 (left) and element2 (right), for all visceral arches, moving rostrocaudally, from the mandibular arch to branchial arch #5, from the bottom to the top of the figure. Left and right elements are shown for Shark#1, #2 and #4 in columns (visual color‐coded key for specimens at top of image). [file AR-309-2262-s003.tif]

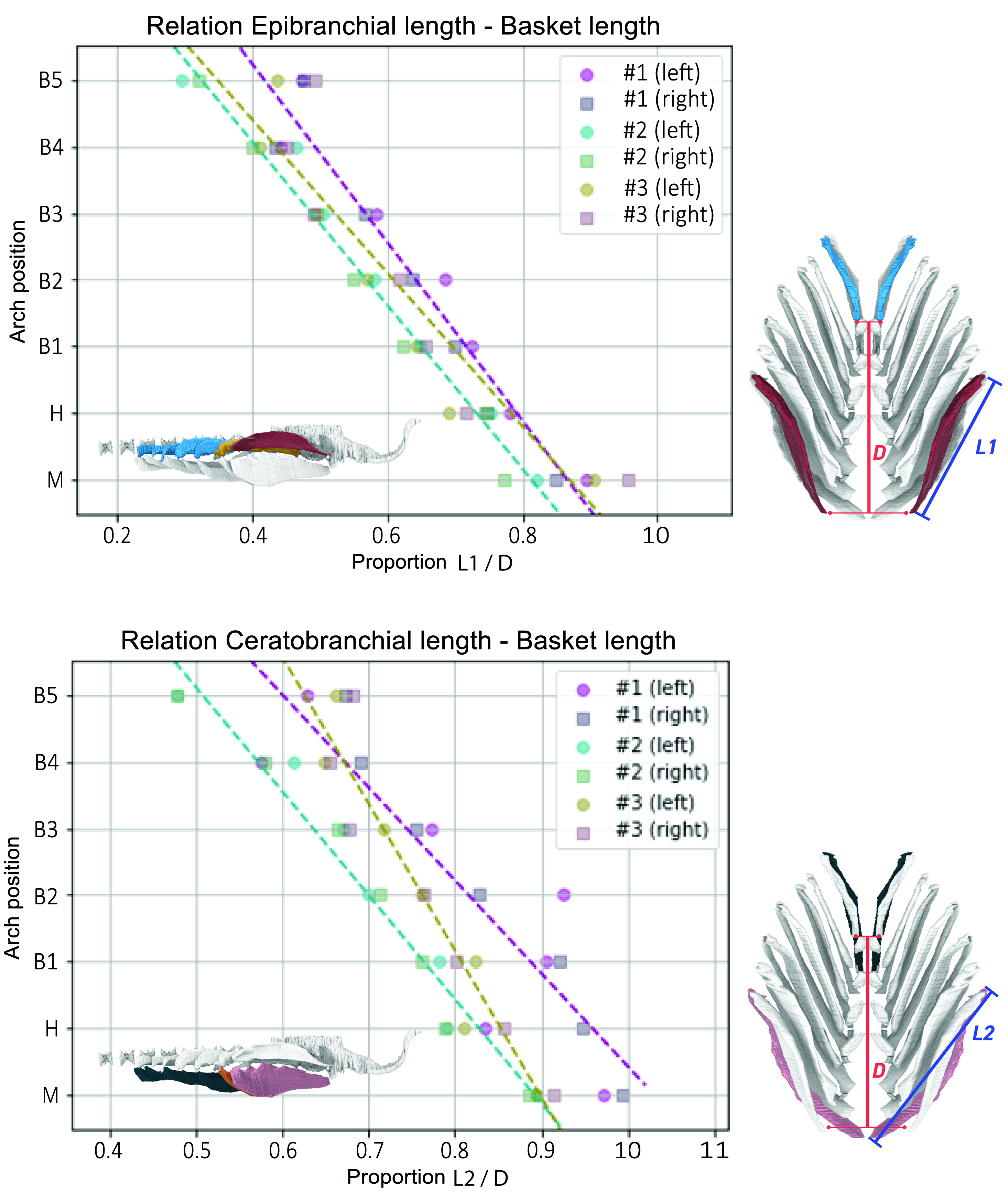

Supplement: Supplementary file 2 — FIGURE S2: Despite variation in animal size, elements have a similar shape. When length of elements is scaled in the graphs relative to branchial basket length (a proxy for body size, not expected to change with mouth movement and distortion of specimens; Figure 8), relative element length is similar across specimens (note the consistent slopes). Since relative element length (i.e., the distance between joints) is key for the biomechanics of our models (Figures 5 and 8), this indicates that our determined model architecture is applicable across specimens. Note the increase in relative arch element length in more rostral arches, which contributes to the conical shape of the pharynx, once open and expanded. [file AR-309-2262-s001.tif]
