# Supplementary material for: Functional models from limited data: A parametric and multimodal approach to anatomy and 3D kinematics of feeding in basking sharks (Cetorhinus maximus)
Source: Anat Rec (Hoboken). 2025 Jun 9;309(9):2262–85. doi: 10.1002/ar.25693 (PMC13431931; doi:10.1002/ar.25693)
Supplement: Supplementary file 3 — TABLE S1: List of specimens used in the current study; see Figures 1 and 2 in the main text for images of each. Information on specimens and their analyses are provided, including notes on specimen condition. [file AR-309-2262-s002.docx]

**Table S1:** List of specimens used in the current study; see Figures 1 and 2 in the main text for images of each. Information on specimens and their analyses are provided, including notes on specimen condition.

| **Shark #** | **Accession number** | **Location/**  **beaching infos** | **Analyses** | **Specs** | **Notes** | **Source** |
| --- | --- | --- | --- | --- | --- | --- |
| Shark 1 | BMNH_1978.6.22.1 | British Museum of Natural History | CT | Resolution:  976 x 976 x 700 µm  Recon: Amira software | Young animal, intact head;  Scanned Royal Brompton Hospital | Kamminga et al., 2017 |
| Shark 2 | BMNH_2004.4.15.30 | British Museum of Natural History | CT | Resolution:  976 x 976 x 700 µm  Recon: Amira software | Young animal, intact head;  Scanned Royal Brompton Hospital | Kamminga et al., 2017 |
| Shark 3 | MCZ_54413 | Museum of Comparative Zoology, Harvard University | CT,  physical examination | Resolution:  976 x 976 x 600 µm  Recon: Amira software | Young animal, intact head;  Scanned Royal Brompton Hospital | Performed by authors |
| Shark 4 | ZMUC_P2401427 | Zoological Museum, University of Copenhagen | CT  physical examination | Resolution:  977 x 977 x 600 µm  Recon: Amira software | Young animal, intact head;  Scanned at Aarhus University Hospital | Performed by authors |
| Shark 5 | ZMUC_P2401428 | Zoological Museum, University of Copenhagen | CT  physical examination | Resolution:  828 x 828 x 600 µm  Recon: Amira software | Young animal, intact head;  Scanned at Aarhus University Hospital | Performed by authors |
| Shark 6 | ZIK  ID: 24840 | Zoological Institute, Kiel University;  found at Hohwacht Bay, Germany, 1983 | photogrammetry |  | Young male animal of 3.75m, cranial skeleton, 5th Branchial arch missing, mounted on metal support frame, Scanned at Kiel University | Performed by authors |
| Shark 7 | NMV_A31308 | Museums Victoria | surface scan and physical exam | 2015: Artec Eva hand scanner  2023: Artec LEO hand scanner | Adult, wet specimen of intact head and fins. Head is scanned by Museum Victoria first time on 2015 with mouth open, and again on 2023 with mouth closed  <https://collections.museumsvictoria.com.au/specimens/2126424> | Museums Victoria |
| Shark 8 | NMV_A19833 | Museums Victoria | Physical examination |  | Young animal, wet specimen of jaws, teeth, gill arches, vertebrae  <https://collections.museumsvictoria.com.au/specimens/104901> | Performed by authors |
| Shark 9 | NMINH: 2013.26.51 | National Museum of Ireland | Micro CT scan, physical examination | Resolution:  arch1_part1:  67.22 x 67.22 x 67.22 µm  arch1_part2:  67.24 x 67.24 x 67.24 µm  Recon: Amira software | Young animal, dried specimens of complete arches with rakers attached | Performed by authors |
| Shark 10 | n/a | YouTube | Photogrammetry, feeding observation | Recon:  Premier Pro,  Agisoft Metashape Professional | Free-swimming adult  **Video:**  Breaching Basking Sharks \| World's Weirdest (0:04-0:11/2:07) @NatGeoWild, youtube <https://www.youtube.com/watch?v=zsC61g36EqM&t=7s> | Photogrammetry performed by author |
| Shark 11 | n/a | YouTube | Photogrammetry, feeding observation | Recon:  Premier Pro,  Agisoft Metashape Professional | Free-swimming adult  **Video:** Solstice - Scotland's Basking Sharks (0:24-0:28/5:00) @simonspear, youtube <https://www.youtube.com/watch?v=aX6xEjOMxys> | Photogrammetry performed by author |
| Shark 12 | ID:BS23042021  [www.iwdg.ie](http://www.iwdg.ie) | Beached at Inchydoney, Cork, Ireland (23/04/21) | Dissection |  | Beached adult female, intact | Performed by authors |
| Shark 13 | ID: BS15052022  [www.iwdg.ie](http://www.iwdg.ie) | Beached at Garryvoe Ballycotton Bay, Cork, Ireland  (15/05/2022) | Anatomical (skeletal) observations from photos |  | Beached adult, skeleton exposed after natural degradation | Performed by Irish Whale & Dolphin Group (IWDG) |
| Shark 14 | ID: BS30042022  [www.iwdg.ie](http://www.iwdg.ie) | Beached at Powerhead, Cork, Ireland (30/04/22) | Dissection |  | Beached adult female, intact | Performed by Frances Gallagher (IWDG) |
| Shark 15 | n/a | Customized Animal Tracking Solutions (CATS) video tags | Feeding observations |  | Free-swimming adults | Cade et al., 2021; Chapple et al., 2024 |
